# NAD Analogs in Aid of Chemical Biology and Medicinal Chemistry

**DOI:** 10.3390/molecules24224187

**Published:** 2019-11-19

**Authors:** Anais Depaix, Joanna Kowalska

**Affiliations:** Division of Biophysics, Institute of Experimental Physics, Faculty of Physics, University of Warsaw, Pasteura 5, 02-093 Warsaw, Poland; anais.depaix@fuw.edu.pl

**Keywords:** nicotinamide-adenine dinucleotide, probe design, synthetic analogs, inhibitors

## Abstract

Nicotinamide adenine dinucleotide (NAD) serves as an essential redox co-factor and mediator of multiple biological processes. Besides its well-established role in electron transfer reactions, NAD serves as a substrate for other biotransformations, which, at the molecular level, can be classified as protein post-translational modifications (protein deacylation, mono-, and polyADP-ribosylation) and formation of signaling molecules (e.g., cyclic ADP ribose). These biochemical reactions control many crucial biological processes, such as cellular signaling and recognition, DNA repair and epigenetic modifications, stress response, immune response, aging and senescence, and many others. However, the links between the biological effects and underlying molecular processes are often poorly understood. Moreover, NAD has recently been found to tag the 5′-ends of some cellular RNAs, but the function of these NAD-capped RNAs remains largely unrevealed. Synthetic NAD analogs are invaluable molecular tools to detect, monitor, structurally investigate, and modulate activity of NAD-related enzymes and biological processes in order to aid their deeper understanding. Here, we review the recent advances in the design and development of NAD analogs as probes for various cellular NAD-related enzymes, enzymatic inhibitors with anticancer or antimicrobial therapeutic potential, and other NAD-related chemical biology tools. We focus on research papers published within the last 10 years.

## 1. Introduction

**Figure 1:**
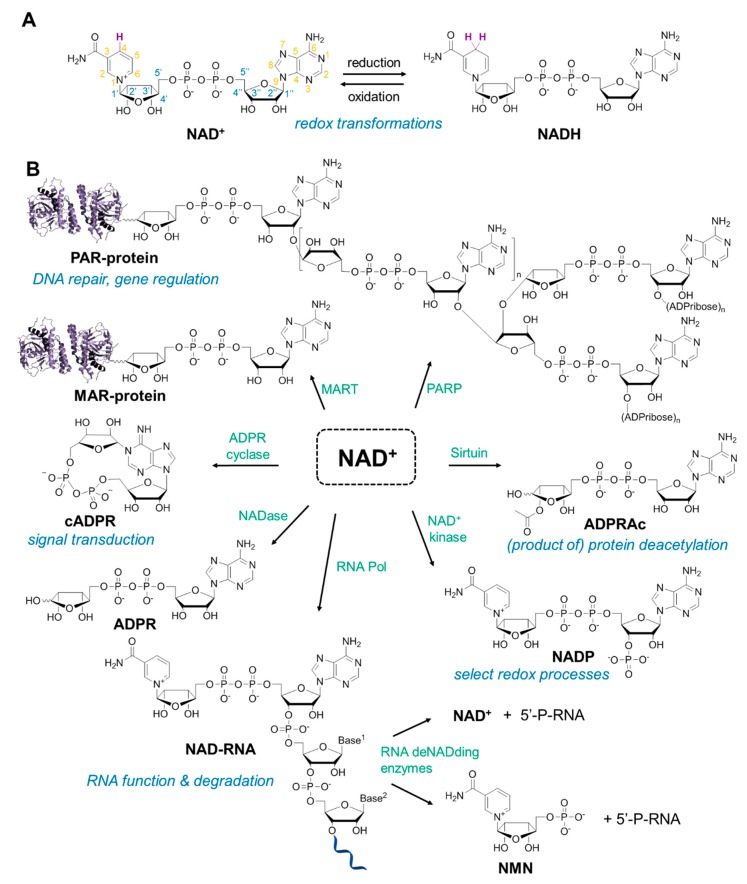
Biological roles of NAD in eukaryotes. (**A**) The NAD^+^/NADH pair commonly involved in cellular redox transformations; atom numbering is shown on NAD^+^; (**B**) Select enzymatic reactions consuming NAD^+^ leading to miscellaneous NAD derivatives and metabolites (labelled in bold). The enzymes catalyzing NAD-dependent reactions are labelled in green and the biological processes involving or affected by their products are marked in blue.

NAD is one of the most important and ubiquitous cofactors present both in prokaryotic and eukaryotic organisms. The essential role of NAD is the participation in cellular redox processes maintained by NAD^+^/NADH interconversion (Figure 1A). NAD can be phosphorylated by NAD kinases to NADP, which is another redox cofactor utilized in biosynthetic pathways and protection against oxidants [1]. In addition to the role in enzyme-catalyzed electron-transfer reactions, NAD is utilized by various enzymes to modify cellular biopolymers or produce signaling molecules (Figure 1B) [2,3,4]. For example, Poly (ADP-ribose) polymerases (PARPs) (also referred to as ADP-ribosyl transferases, ARTs) use NAD^+^ to post-translationally modify proteins by addition of poly-ADP-ribose (PAR) chains in response to DNA damage. PARP inhibitors have potential as anticancer and chemo-sensitizing drugs [5,6]. Another class of disease-associated NAD-dependent enzymes are sirtuins, which utilize NAD^+^ to remove acyl groups from proteins such as histones and transcription factors, thereby regulating gene expression [7]. Some sirtuins, along with other enzymes, possess mono-ADP-ribosyltransferase activity. Cyclic ADP-ribose (cADPR), one of the key secondary messengers in calcium signaling, is produced from NAD^+^ by ADP-ribosyl cyclase (CD38 family), which is an ectoenzyme involved, among others, in immune response and cell adhesion. CD38 has also NAD glycohydrolase activity (referred to also as NAD nucleotidase or NADase), i.e., it catalyzes NAD^+^ hydrolysis through glycosidic bond cleavage leading to release of nicotinamide (Nam) and ADP-ribose, thereby additionally contributing to NAD turnover and signaling pathways. NAD is also a target for intracellular hydrolases, such as bacterial NudC or mammalian NUDT12 pyrophosphatases, which contribute to the control of endogenous NAD levels [8]. Most recently, NAD has been found to serve as a ‘metabolite cap’ present at the 5′ end of some cellular RNAs in various organisms, from bacteria to higher mammals [9,10,11,12,13,14,15,16,17]. The modification is introduced to RNA co-transcriptionally by RNA polymerases or synthesized de novo using nicotinamide mononucleotide (NMN) [18,19,20,21]. Although the biological function of NAD-capped RNAs remains largely unclear, an increasing number of literature reports indicate that NAD tag strongly influences RNA stability and is targeted by specific cellular hydrolases [22,23]. 

This multitude of NAD-dependent molecular processes orchestrates a variety of complex biological functions controlling DNA repair, cell survival and apoptosis, response to stress, circadian rhythm, aging and longevity, immune response, and many others [2,3,24,25,26,27]. Consequently, there is a strong demand for NAD-derived molecular tools that could aid the efforts towards deeper understanding of these biological pathways as well as prompt the development of new therapeutic strategies. Indeed, many synthetic NAD analogs have been designed as reporter substrates or modulators for NAD-dependent enzymes with therapeutic potential. For instance, the inhibition of anabolic pathways leading to generation of NAD and NADP is explored as an anti-cancer and antimicrobial strategy, whereas activation of those pathways may be a viable protective strategy to mitigate oxidative damage and age-related degenerative diseases. 

Here, we review the recent advances in the development of molecular tools and therapeutic candidates derived from or inspired by the NAD structure. To this end, we focus on five main areas that, in our opinion, have been the driving force for the development of NAD analogs in the past decade:

- NAD analogs as fluorescent probes for real-time monitoring of miscellaneous NAD-processing enzymes
- NAD analogs for the detection and visualization of polyADPribose polymerase activity
- Potential therapeutic agents derived from NAD structure
- NAD analogs for the development of biorthogonal redox systems
- Methods and tools to advance our understanding of NAD-capped RNAs

We conclude our review by providing a short personal perspective on the future challenges and opportunities for the field. 

## 2. Fluorescent NAD Analogs for Real-Time Monitoring of NAD-Processing Enzymes

Cofactors and cofactor analogs with distinctive optical properties have paved the way for many important applications, including monitoring enzymatic transformations in real-time, developing inhibitor discovery assays, and studying cofactor-related covalent protein modifications in vitro and in vivo. Both NAD^+^ and NADH absorb in the UV region, but differ in absorptive and emissive properties. NAD^+^ absorbs with a single maximum around 260 nm, whereas NADH has additionally a second absorption band with maximum around 340 nm corresponding to the reduced form of nicotinamide riboside. Moreover, NADH has emissive properties with maximum at 460 nm, while NAD^+^ is virtually non-fluorescent. These differences in spectroscopic properties have been widely applied to monitor the enzymatic activity of NAD-dependent redox enzymes. The changes in fluorescent properties of NADH have also been exploited to study protein binding in vitro and in live cells [28,29,30]. However, the relatively low quantum yield of NADH (~2%) [31] and lack of emission for NAD^+^ exclude applications in the investigation of many other NAD-related phenomena. Therefore, there is a strong demand for the development of fluorescent NAD analogs that can act as NAD surrogates in various biological settings. 

**Figure 2:**
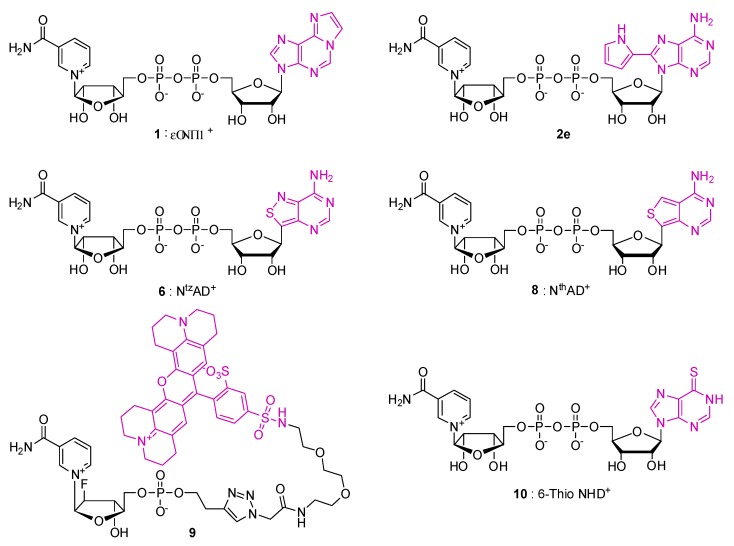
Representative NAD^+^ analogs developed as fluorescent probes for NAD-consuming enzymes. The fluorophore is marked in purple. For emissive properties, see Table 1.

An important class of NAD-derived molecular tools are analogs with photophysical properties responsive to structural changes occurring during miscellaneous enzymatic transformations. These analogs are usually modified by replacing adenosine with adenosine analog exhibiting emissive properties. The first analog representing this group, developed as early as the 70s, was ε-NAD^+^ (**1**) containing 1,N6-ethenonoadenosine (ε-A, Figure 2). However, the analog poorly supported many of the studied NAD-dependent processes (Table 1), which was explained by the bulkiness and significantly altered H-bonding pattern of ε-A. Hence, the use of ε-NAD^+^ **1** was limited to enzymes for which purine ring recognition is not critical for binding at the active site. During the last decade, a significant surge has been observed in adenosine-modified NAD analogs with properties superior to ε-NAD. 

**Scheme 1:**
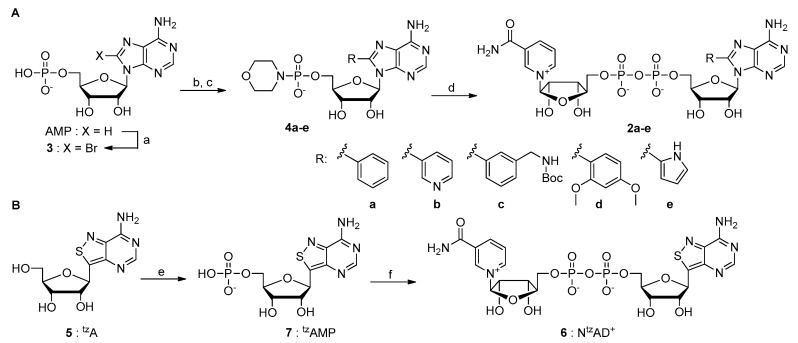
Chemical synthesis of select fluorescent NAD^+^ analogs [32,33]. (**A**) 8-substituted NAD^+^ analogs reported by Pergolizzi et al. and (**B**) N^tz^AD^+^ first reported by Rovira et al. Conditions: a) Br_2_, KH_2_PO_4(aq)_, rt; b) Na_2_PdCl_4_, TPPTS, R-B(OH)_2_, K_2_CO_3_, H_2_O, Δ, 1–24 h; c) morpholine, dithiodipyridine, Ph_3_P, DMSO, rt, 2 h; d) NMN, dry MgSO_4_, MnCl_2_, formamide, rt, 24 h; e)1. POCl_3_, proton sponge, (MeO)_3_PO, 4 °C, 2 h, 2. hydrolysis; f) NMN imidazolide (synthesized from NMN using CDI, Et_3_N, DMF at rt), DMF, rt, 4 days.

Pergolizzi et al. [32] reported a series of NAD analogs containing an aromatic fluorogenic substituent at the 8-position (Scheme 1A, compounds **2a**–**e**). The substituents were introduced by derivatization of 8-Br-adenosine monophosphate **3** (8-Br-AMP) under Suzuki–Miyaura aqueous coupling conditions. The dinucleotides were then obtained by coupling the corresponding 8-substituted AMP morpholidate **4** with NMN in formamide in the presence of MgSO_4_ and MnCl_2._

The fluorescence properties of these compounds varied depending on the nature of the 8-substituent, but in all cases, the fluorescence quantum yield for NAD analog was lower than that for the corresponding AMP analog, indicating intramolecular quenching effect in NAD caused by stacking of nucleobases, similar to that previously described for ε-NAD. This effect was particularly strong for compound **2e** (46-fold quantum yield increase upon removal of nicotinamide moiety). Thus, the utility of this analog to study NAD-consuming processes was verified in the context of three enzymatic activities: glycohydrolase (from porcin brain), pyrophosphatase (snake venom phosphodiesterase from *Crotalus adamanteus*), and ADP-ribosyl cyclase (ADPRC from *Aplysia californica*), which also possess N1-cADPR hydrolase activity. Analog **2e** was readily consumed by pyrophosphatase and glycohydrolase activities, which, as expected, resulted in a strong increase of fluorescence intensity, confirming its utility as a turn-on probe for enzymatic activity monitoring. The behavior of **2e** towards ADPRC depended on enzyme concentration. In the presence of low enzyme concentration, the decrease of fluorescence intensity was observed and interpreted as a result of cyclization of NAD analog at the N1-position, to give 8-substituted N1-cADPR. At higher enzyme concentrations after initial fluorescence drop, fluorescence increase was observed, which was interpreted as the hydrolysis of N1-cADPR into ADPR analog. Hence, the new NAD analog **2e** allowed direct visualization of the different reaction pathways catalyzed by ADPRC. 

Significant advancements in the field of fluorescent NAD analogs for enzymatic activity monitoring have been achieved by the Tor group by implementation of their unique adenosine analogs, isothiazolo[4,3-d]pyrimidine riboside (abbreviated as ^tz^A, **5**, Scheme 1B) and thieno[3,4-d]pyrimidine riboside (abbreviated as ^th^A). These nucleosides have initially been developed as tools for oligonucleotide modification and functional probes for ATP-consuming enzymes and are shown to minimize the structural and functional perturbations arising by replacing adenosine due to very similar size and H-bonding pattern [34,35,36,37]. 

Rovira et al. [33] incorporated ^tz^A moiety into NAD to obtain an isomorphic and isofunctional analog **6,** enabling real-time monitoring of various NAD transformations. N^tz^AD^+^ (**6**) was synthesized starting from ^tz^A (**5**), which was treated with phosphorus oxychloride (POCl_3_) in trimethyl phosphate to yield ^tz^AMP (**7**), which, in turn, was coupled with imidazole-activated NMN (obtained by treatment with CDI) in DMF (Scheme 1B). 

The spectroscopic properties of N^tz^AD^+^ were very similar to those of isolated ^tz^A with emission maximum at 411 nm (Table 1). The biocompatibility of N^tz^AD^+^ and the sensitivity of fluorescence properties to structural transformations were tested with several enzymes, including dehydrogenases, NAD glycohydrolase, and monoADP-ribosyltransferases. N^tz^AD^+^ was converted into N^tz^ADH by *Saccharomyces cerevisiae* alcohol dehydrogenase (ADH) with efficiency comparable to that of NAD^+^. About three-fold decrease in fluorescence intensity was observed during this process, making the properties of this analog complementary to those of NAD^+^ (for which fluorescence intensity increases upon reduction). The N^tz^ADH process could be almost completely reversed by addition of either acetaldehyde or lactate dehydrogenase (LDH) and pyruvate. Both N^tz^AD^+^ and N^tz^ADH were hydrolyzed by porcine brain glycohydrolase showing modest (30–40%) increase in fluorescence. Finally, the emissive properties of N^tz^AD^+^ were employed to real-time monitoring of protein monoADPribosyltransferase (MART) activity. In this context, two MARTs were tested in the presence of agmantine as a model substrate, *human* ART5 and cholera toxin subunit A (CTA). It was found that the human enzyme catalyzed almost exclusively hydrolysis of NAD^+^/N^tz^AD^+^ to the corresponding ADPribose, whereas CTA primarily catalyzed ADPribosylation of agmantine. Both processes could be efficiently monitored by emission spectroscopy if N^tz^AD^+^ was used as a substrate. Halle et al. [38] aimed to increase the availability and applicability of N^tz^AD^+^. To this end, they developed an enzymatic method for the synthesis of N^tz^AD^+^ from ^tz^ATP and NMN catalyzed by nicotinamide mononucleotide adenylyl transferase 1 (NMNAT-1). Further, they showed that N^tz^AD^+^ can be phosphorylated by NAD^+^ kinase (NADK from *B. subtilis*) to the corresponding N^tz^ADP^+^, which in turn serves as a substrate for glucose-6-phosphate dehydrogenase (G6PDH from *S. cerevisiae*) producing N^tz^ADPH. Finally, N^tz^ADPH can be reoxidated to N^tz^ADP^+^ by glutathione reductase. The emissive properties of the N^tz^ADP^+^/N^tz^ADPH redox system were very similar to those previously reported for N^tz^AD^+^/ N^tz^ADH pair and enabled real-time monitoring by emission spectroscopy. 

Feldman et al. [39] applied a similar chemoenzymatic approach to obtain a thieno[3,4-d]pyrimidine-based NAD^+^ analog (N^th^AD^+^, **8**, Figure 2). Synthetic ^th^ATP was quantitatively converted into N^th^AD^+^ by NMNAT1 in the presence of NMN, albeit at a lower rate than ATP or ^tz^ATP. 

N^th^AD^+^ showed slightly red-shifted emission and almost 2-fold higher brightness compared to N^tz^AD^+^. The compound served as a substrate for alcohol dehydrogenase (from *S. cerevisiae*) producing N^th^ADH, but the process was accompanied by only 8% decrease in fluorescence. The analog was also a substrate for porcine brain glycohydrolase producing ^th^ADPR. The kinetics for this analog was again slower than in the case of N^tz^AD^+^, yet provided about four-fold higher sensitivity. Qualitatively similar observations were made for CTA-catalyzed ADP-ribosylation of agmantine. Finally, both N^tz^AD^+^ and N^th^AD^+^ were tested as substrates for human polyADPribosyltransferase (PARP1) in auto-polyADPribosylation assay. It turned out that N^th^AD^+^ is a much better substrate for PARP1 than N^tz^AD^+^, but none of the analogs enabled real-time monitoring of the process. All in all, the results indicated that despite higher quantum yield and responsiveness of N^th^AD^+^ relative to N^tz^AD^+^, the lack of N7-nitrogen atom causes reduced compatibility with biological processes. The optical and biological properties of select fluorescent NAD analogs are summarized in Table 1.

Other types of fluorescent NAD analogs, usually tailored for a single specific application, have also been described. A rhodamine-labelled NMN analog (compound **9**, Figure 2) that was employed as a probe for covalent labelling and real-time dynamics monitoring of the CD38 receptor (which possesses NAD glycohydrolase activity) has been reported by Schrimp et al. [43]. Moreau et al. [44] synthesized a nicotinamide 6-mercaptopurine 5′-dinucleotide (6-Thio NHD^+^, **10**, Figure 2) and found that it is accepted as a substrate by ADP-ribosyl cyclase to form a fluorescent N1-cADPR analog with emission maximum at 415 nm. Fluorescence methods have also been widely explored to study protein mono- and polyADP ribosylation, including real-time visualization [45]. Yet, in this context, the post-enzymatic fluorescent labelling of modified PAR-protein, obtained using an appropriately functionalized NAD analog, is the commonly adopted approach; therefore, we will review this topic separately in the next paragraph. 

## 3. NAD Analogs to Study Protein ADP-Ribosylation

ADP-ribosylation is a post-translational protein modification catalyzed by ADP-ribosyltransferases (ARTs), which regulates DNA repair and gene expression. The reaction comprises of the transfer of either single (mono-ADPribosylation) or multiple (polyADPribosylation or PARylation) moieties on specific amino acid residues in the target protein, catalyzed by MARTs and PARPs, respectively. Different amino acids with nucleophilic groups in the side chains have been identified as modification sites, including glutamate, aspartate, arginine, lysine, serine, and cysteine. The polyADPribose chains are added onto the target proteins upon DNA damage to trigger repair mechanisms. The growing polymer consists of either linear or occasionally branched chains consisting of up to a few hundred ADP ribose subunits [46], which makes analysis of PARylated proteins a challenging task. Mono-ADPribosylation leads usually to the inactivation of the target protein and is catalyzed not only by intracellular enzymes, but also by bacterial toxins [47]. 

**Figure 3:**
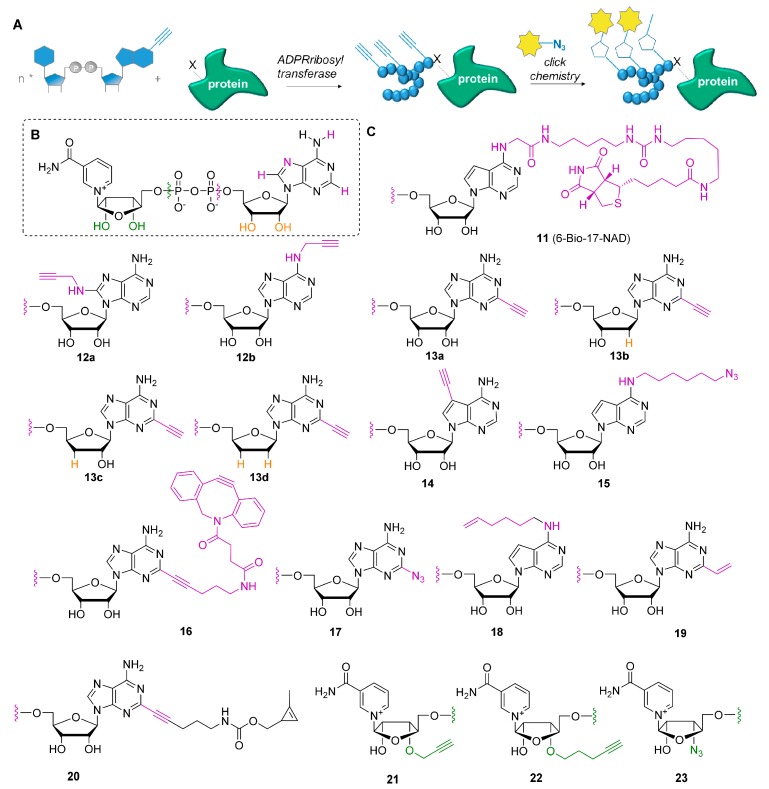
NAD^+^ analogs used as substrates for protein ADP-ribosylation studies. (**A**) The idea of chemoenzymatic ADPR-ribose labelling using modified NAD^+^ analogs; (**B**) NAD^+^ modification sites (in purple, orange, and green) explored in the literature so far; (**C**) overview of analogs tested in this application.

One of the approaches to study protein targets for mono- and poly-ADP-ribosylation is by taking advantage of chemically modified substrates that are utilized by enzymes similarly as NAD^+^ and are either already labelled or amenable to post-enzymatic labelling with fluorescent dyes or affinity tags (Figure 3A). A ground-laying work for this field was reported by Zhang [48], who developed NAD^+^ analogs labelled with biotin and tigitoxin at positions 6 or 8 using NHS chemistry. The most promising analog (6-Bio-17-NAD **11**; Figure 3B) was initially validated by labelling of Elongation Factor 2 through diphtheria toxin-catalyzed ADP-ribosylation and affinity purification of nitric oxide-enhanced ADP-ribosylated protein [48], and later was employed by others to study PARP activation in cells and tissues under oxidative stress or diabetic dysfunction [49]. More recently, NAD^+^ analogs modified with an alkyne or azide group have been introduced and can be easily functionalized using azide-alkyne cycloaddition (AAC) click reaction. Du et al. [50] investigated mono ADP-ribosylation catalyzed by sirtuins using alkyne functionalized NAD^+^ analogs. To this end, two NAD^+^ analogs, containing a propargyl moiety either at the position C8 or N6 position (Figure 3, compounds **12a** and **12b**), were synthesized using diphenyl phosphoryl chloride activation for the pyrophosphate bond formation and tested as substrates for sirtuin catalyzed-ADP ribosylation in cell extracts and in vitro. The monoADP-ribosylated products were detected and isolated by reacting them with azido-functionalized and biotin-coupled rhodamine. The results indicated that the tested analogs of NAD^+^ are substrates for sirtuins, which was independently confirmed by ^32^P-NAD^+^. However, the ADP-ribosylation process had slow kinetics, which led the authors to believe that it is a non-physiological artifact, rather than a physiologically relevant process. Jiang et al. [51] used the same set of NAD^+^ analogs to identify substrate proteins of PARP-1 (known also as ARTD1) and Tankyrase-1. To that end, they first investigated substrate properties of the compounds with recombinant enzymes, followed by experiments in cell extracts. Both PARP-1 and Tankyrase-1 underwent PARylation in the presence of these analogs, as shown using SDS-PAGE analysis. However, the NAD^+^ analog labelled at the N6 position (**12b**), with *k*_cat_/*K*_M_ value 12- (PARP-1) and 4-fold (Tankyrase-1) lower compared to NAD^+^, was a much better substrate that the one labeled at the C8-position (**12a**). 6-alkyne NAD^+^ was then incubated in cell extracts, followed by chemical reaction with azido-biotin and protein isolation on streptavidin beads, which led to the isolation of 79 ADP-ribosylated proteins. These included known PARP-1 targets, thereby validating the method, as well as potential novel ones. Proteins polyADPribosylated with clickable NAD^+^ analogs are also often analyzed by electrophoresis combined with fluorescent labelling of PAR moieties by copper catalyzed AAC (CuAAC) for visualization. However, the differences in the composition of the synthesized PAR chains, which may differ in length and branching pattern, result in variable migration and pose difficulty in the analysis of complex mixtures of PAR-proteins. 

The Marx’s group has proposed several strategies to overcome this difficulty, without compromising the robustness of the PARylation analysis. Wang et al. [52] designed NAD^+^ analogs that carried an alkyne moiety at the C2 position of adenosine to enable derivatization by CuAAC and optionally were modified at the ribose moiety of adenosine to make them substrates that acted as chain terminators for polyADPribosylation reaction (Figure 3, compounds **13a**–**d**). The synthesis of the analogs **13a**–**d** was achieved from appropriately modified 2-iodoadenosine derivatives, followed by a Sonogashira coupling with trimethylsilylacetylene, phosphorylation with POCl_3_, and coupling with CDI-activated NMN. 

**Figure 4:**
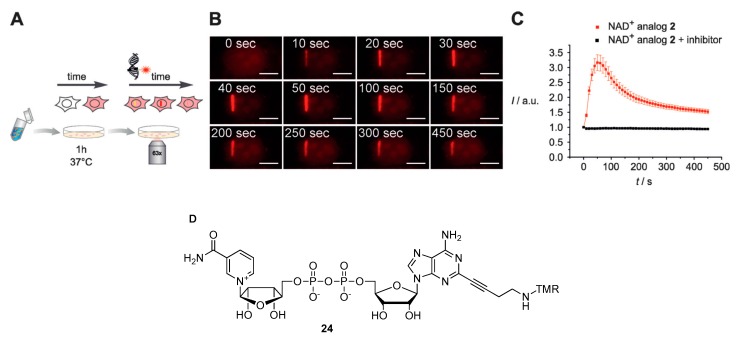
Real-time monitoring of PAR turnover in living cells using NAD^+^ analog **24**, labelled with tetramethylrodamine (TMR). (**A**) Experiment overview. (**B**) Time-dependent snapshots of cell nucleus obtained by confocal microscopy. The site-specific DNA damage was induced at 0 sec by microirradation at 775 nm. (**C**) Quantitative data obtained from multiple experiments. (**D**) Structure of compound **24**. A–C are reproduced with permission from Buntz et al., *Angew. Chem. Int. Ed. 55*, 11256; published by John Wiley and SONS, 2016 [45].

The compounds were then tested as substrates for ARTD1 in auto-ADP-ribosylation reaction as well as in trans-ADP-ribosylation of histone H1.2. As expected, compounds **13b** and **13d** lacking the 2′’-hydroxyl group or both 2′’- and 3′’-hydroxyls, respectively, were accepted as substrates for ARTD-1, but produced much shorter products, compared to compound **13a** with unmodified ribose moiety. Importantly, the products obtained with analogs **13b** and **13d** migrated as a single band during electrophoretic analysis. The compound lacking only the 3′’-hydroxyl (**13c**) resulted in more efficient and heterogeneous PAR formation, albeit still less efficient than the ribose-unmodified analog **13a**. Moreover, it was demonstrated that the dideoxy compound **13d** efficiently competed with NAD^+^ in trans-ADP-ribosylation reaction, but not for auto-ADP-ribosylation. The proteins modified with these analogs were not only visualized by azido-Cy5 but also affinity tagged with azido-biotin, highlighting the possibility of application in proteomic analyses. Wallrodt et al. [53] aimed to apply functionalized NAD^+^ to intracellular imaging of ARTD-1 catalyzed PARylation. To that end, they explored both known and new NAD^+^ analogs creating a set of compounds varying in the position of modification (N6-, and C2 or C7) and the functional group type enabling labelling through different bioorthogonal reactions (copper catalyzed or strain promoted azide-alkyne cycloaddition, CuAAC and SPAAC, respectively, and inverse electron demand Diels-Alder cycloaddition, IEDDA) (Figure **3,** compounds **14**–**20**). First, the variously functionalized analogs were tested in the previously established in vitro assay using histone H1.2, to gain insight into the substrate scope of ARTD-1. It was found that in contrast to modifications at the positions N6 and C2 of adenosine, the presence of the ethynyl substituent at the C7 position of C7-deazaadenosine completely abolished PAR formation. The analogs modified at the N6-position with bulkier substituents (**15** and **18**) supported PAR formation, but only in the presence of NAD^+^, indicating that they are not good initiators of this process. The C2 position of adenosine was found to be most compatible with ARDT-1 as even analogs carrying bulky substituent enabling SPAAC (**16**) or IEDDA (**19**, **20**) efficiently supported PAR formation both in the presence and absence of NAD^+^. Finally, it was found that the modified PAR chains can be degraded by PARG (poly(ADP-ribose) glycohydrolase), the major PAR degrading enzyme, which leads to formation of mono-ADPribosylated proteins, thereby simplifying their analysis. These analogs were next tested in cultured cells, to select compounds suitable for visualization of nuclear protein PARylation occurring after oxidative DNA damage induced by H_2_O_2_ treatment. The NAD^+^ analogs were incubated with transiently permeabilized cells, followed by cell fixing, labeled with dyes containing complementary functional groups, and analysis by confocal microscopy. Among the tested analogs, the highest signal-to-noise ratios, indicating for the highest specificity, were obtained for compounds **13a** and **16** functionalized with alkyne at the C2-position that are amenable to labelling with azide-functionalized dyes using CuAAC and SPAAC. In the final experiment, it was demonstrated that by the simultaneous use of analogs **13a** and **16,** two-color labelling of PAR moieties in cells is possible, revealing potential of these compounds for applications in more advanced experimental techniques. Fluorescent NAD^+^ analogs as probes for real-time monitoring of PAR formation in cells have been developed by Buntz et al. [45] (Figure 4). To that end, two NAD^+^ analogs, labelled at the N6 or C2 position with TMR, were synthesized. In vitro evaluation revealed, similarly as in other studies, that the C2 substituted compound **24** (Figure 4D) was more competitive and efficient substrate for ARTD-1. The analogue **24** was successfully used in real-time monitoring of PAR synthesis and degradation in living cells for the first time. (Figure 4A–C). Moreover, using cells expressing GFP-fused ARTD1 and FLIM-FRET microscopy, the interaction of modified PAR polymer and specific protein in living cells was demonstrated.

Recently, another group has for the first time explored the modifications of nicotinamide riboside for protein PARylation studies [54]. To that end, Zhang et al. reported a set of three NAD^+^ analogs modified with either an alkyne or azide group at the 3′-position of nicotinamide riboside (Figure 3, **21**–**23**) and their 2′-counterparts (not shown), which are accessible by either chemical or chemoenzymatic methods. Preliminary characterization identified compound **23** (3′-N_3_-NAD^+^) as the most active and highly specific substrate of PARP1 and PARP2 that has kinetic parameters superior to previously reported adenine-modified compounds and is amenable to post-enzymatic labelling with biotin or fluorescent tags using CuAAC. The utility of compound **23** was demonstrated by in vitro and cell lysate PARylation assays, followed by visualization and labeling of mitochondrial PARylated proteins in fixed HeLa cells.

## 4. NAD-Derived Inhibitors of Biologically and Therapeutically Relevant Processes

Cancer and infectious diseases such as tuberculosis or malaria are responsible for millions of deaths every year. An increasing problem in treating those diseases is that the long-term use of drugs leads to resistance. This necessitates a continuous search for new active compounds. The development of new drug candidates is often initiated by the design of analogs of natural compounds that could target specific cellular pathways. Since disruption in NAD metabolism can lead to serious consequences, NAD-dependent enzymes are potential antimicrobial or anticancer drug targets. In the next paragraphs, we will review recent efforts to develop NAD-derived inhibitors, which offer new functional and mechanistic insights, as well as NAD-inspired drug candidates, which provide perspectives for fighting disease. 

### 4.1. NAD Analogs Targeting Enzymes that Attack the Glyosidic Bond

#### 4.1.1. CD38 Ligands and Inhibitors

CD38 is a multifunctional glycoprotein expressed on the surface on many types of cells, including immune cells, which regulates various signaling pathways. CD38 acts as a promiscuous ectoenzyme that catalyzes NAD^+^ conversion to cADPR, the hydrolysis of cADPR to ADPR, and the deamidation of NADP^+^ to nicotinic acid adenine dinucleotide phosphate (NaADP), thereby participating in Ca^2+^-dependent signaling. It has been connected to many diseases, and CD38-targeting antibody has been approved as a therapeutic for multiple myeloma [55]. Development of small molecules targeting CD38 has also been pursued to deepen the understanding of it biological functions as well as develop drug candidates. As a natural substrate for CD38, NAD^+^ has been often used as a starting point for inhibitor design.

**Figure 5:**
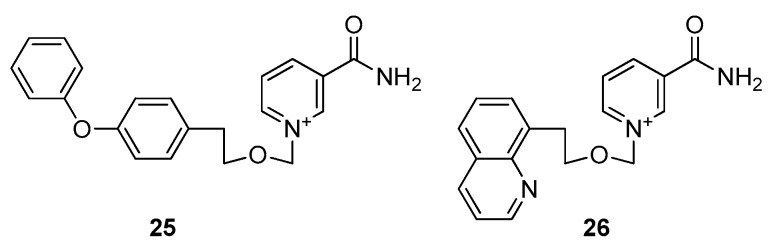
Inhibitors of CD38 NADase activity showing muscle relaxation effect [56].

In 2011, Dong et al. [56] designed a set of 14 analogs, based on a nicotinamide moiety attached to an aromatic substituent via an ether linkage. The linker was introduced in replacement of the pyrophosphate bond present in the natural NAD^+^ substrate, to ensure better bioavailability. The susceptibility of analogs to inhibit NAD glycohydrolase (NADase) activity of CD38 was assessed using an in vitro assay. All of the compounds had only weak inhibitory activity (2 mM < IC_50_ < 10 mM). Nonetheless, an observation was made that the presence of an aromatic group at the distal end of the nicotinamide moiety and a longer aliphatic chain increase the efficiency of the inhibition, whereas the presence of an electron withdrawing group such as CF_3_ leads to the loss of the affinity for CD38. To verify the biological activity of these compounds, the authors assessed their physiological effects on muscle relaxation. Indeed, it has been shown that Ca^2+^ increase is required for airway smooth muscle contraction, which can be induced by the presence of acetylcholine (Ach). This is mediated by CD38, which can, thereby, be used as a target for investigating its physiological role in cells and tissues. Using the tracheal trips from rat/guinea pig, the authors induced contraction of this muscle by Ach treatment and subsequently introduced the inhibitors described above. Among the tested compounds, two (**25** and **26**, Figure 5) have led to a significant relaxation of muscle, in a concentration dependent way. Compound **25** was also crystallized in complex with CD38, confirming that its nicotinamide moiety binds in the active site in a similar way as in NAD^+^, providing a guide for the design of more potent inhibitors of CD38.

**Figure 6:**
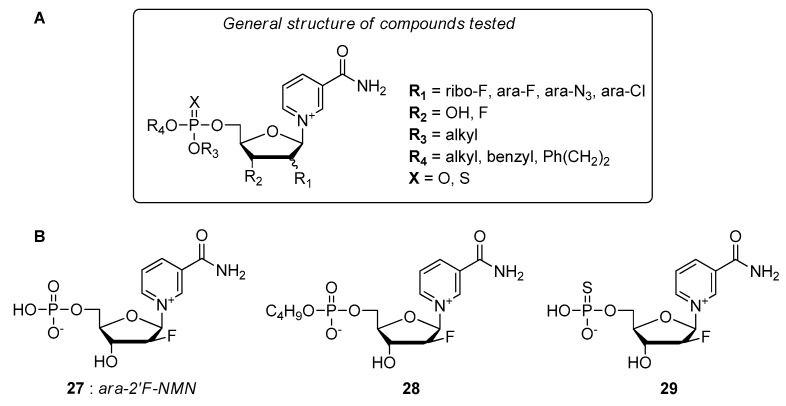
(**A**) General structures of the different NMN derivatives synthesized as CD38 inhibitors; (**B**) structures of ara-2′F-NMN **27** and analogs **28** and **29** [57].

**Figure 7:**
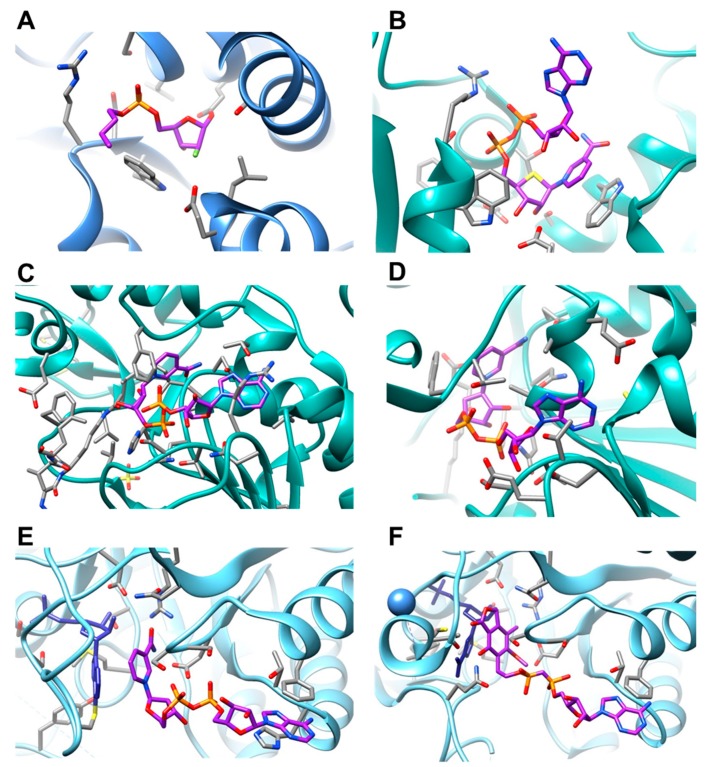
Crystallographic structures of representative NAD^+^ or analogs in complexes with target proteins. (**A**) NAD^+^ analog **28** bound to CD38 (enzyme labelled in blue) PDB code: 3ROK; (**B**) S-NAD^+^ **39** bound to SIRT2 (enzyme labelled in green), PDB code: 6EDR; (**C**) carba-NAD^+^ **33** bound to SIRT3 (enzyme labelled in green), PDB code: 4FVT; (**D**) carba-NAD^+^ **33** bound to SIRT5 (enzyme labelled in green), PDB code: 4G1C; (**E**) NAD^+^ bound to *h*IMPDH2 (enzyme labelled in light blue), PDB code: 1NFB; (**F**) MAD **44** bound to *h*IMPDH2 (enzyme labelled in light blue, ligand labelled in dark blue), PDB code: 1NF7. General labels for all the structures: carbon from NAD^+^ analog in purple, carbon from amino acid side chains in grey, oxygen in red, nitrogen in blue, phosphorus in orange and sulfur in yellow.

In 2012, Kwong et al. [57] synthesized stable NAD^+^ analogs and studied their inhibitory properties towards NADase activity of CD38. As the starting point for the design of inhibitors, they chose ara-2′F-NMN **27** (IC_50_ 0.5 µM, Figure 6), which was known as one of the most potent inhibitors, but was unstable against phosphatase activity. The first modifications that were tested to potentially overcome this difficulty were phosphate substitutions with two alkyl groups (Figure 6A, R3 and R4), but they led to the loss of inhibition. Therefore, in the next iteration, only one alkyl group was introduced, which led to only modest loss of inhibition. Sugar moiety modifications were also explored in this study. The change in orientation of 2′-F(R^1^) did not lead to a significant change in inhibitory effect, whereas introducing 3′-F(R^2^) instead of 2′-F led to dramatic increase in the IC_50_ value (IC_50_ > 0.25 mM). Replacing the fluorine atom by either Cl or N_3_ also increased IC_50_ (IC_50_ = 11 µM and IC_50_> 1 mM, respectively). Finally, by replacing phosphate with thiophosphate (Figure 6A, X = S) or by introducing bulkier aromatic groups onto the phosphate, they obtained compounds with IC_50_ only slightly higher than ara-2′F-NMN **27** (IC_50_ < 3 µM). Compound **28** (IC_50_ 2 µM, Figure 6B) was crystallized with CD38 (Figure 7A), confirming that the negatively charged phosphate is crucial for the interaction with this protein. Overall, the study provided new insights into SAR of ara-2′F-NMN and emphasized the importance of the negative charge on phosphate group for inhibition of the NADase activity of CD38. Importantly, the presence of thiophosphate or monosubstituted phosphate reduced the susceptibility of compounds to phosphatases, thereby increasing their metabolic stability and enabling more advanced biological studies. Hence, the effects of those compounds toward two cADPR-related processes were then studied. cADPR level increases concomitantly with CD38 expression when inducing HL-60 cells differentiation by retinoic acid. Thus, the authors have tested the inhibition effect of compound **28** in cell model and found a concentration-dependent response, leading to the decrease of cADPR levels. It has also been shown that lowering the amount of cADPR in rat lacrimal cells results in impairment in aortic contraction. After confirming that the compound **29** can inhibit rat and human CD38 (IC_50_ 2 µM), it was found that this compound inhibits the contraction of rat mesenteries, previously induced by the addition of phenylephrine, with a half maximal effective concentration of 30 µM. These results confirmed that properly designed CD38 inhibitors are useful tools for studying CD38/cADPR signaling pathways in cells and tissues.

**Figure 8:**
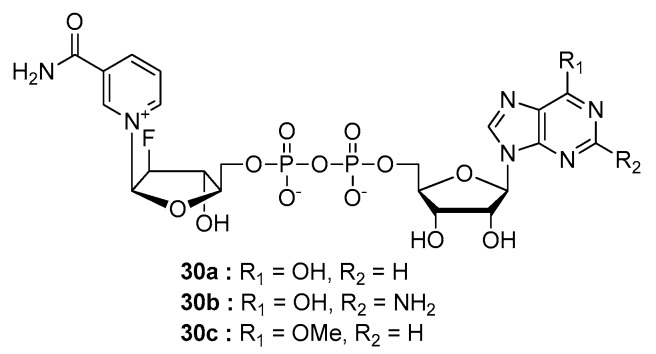
Best potent inhibitors of NADase activity of CD38 described by Wang et al. [58].

Wang et al. [58] approached the development of CD38 inhibitors by exploring various modifications of all three structural parts present in NAD^+^, namely the ribose, the pyrophosphate, and the nucleobase. After in vitro evaluation as CD38 NADase inhibitors, three most active compounds were selected (**30a**–**c**, Figure 8). Interestingly, all of them possess a 2′-*ara* fluorine atom on the nicotinamide moiety as well as purine modification. Other modifications such as replacement of the phosphate neighboring the nicotinamide with thiophosphate, a second 2′-substituent in addition to the 2′-*ara*-F, or the replacement of adenine with nicotinamide resulted in lower inhibitory activity. This revealed the importance of the pyrophosphate bridge and the purine ring for recognition, albeit the latter showing some flexibility towards modification. 

#### 4.1.2. NAD Analogs Targeting Sirtuins and ADPribosyltransferases

Sirtuins are NAD-dependent enzymes, which induce the removal of acyl groups of different lengths (from C2 up to C14) from amino acid residues in various protein substrates, with deacetylation of acetyllysine being the most thoroughly studied process [59]. Human sirtuins can be classified into seven families (SIRT 1–7) and are involved in different cellular processes, including aging, transcription, and apoptosis. Some sirtuins have been identified as molecular targets for disease treatment, including cancer, diabetes, and age-related diseases.

**Scheme 2:**
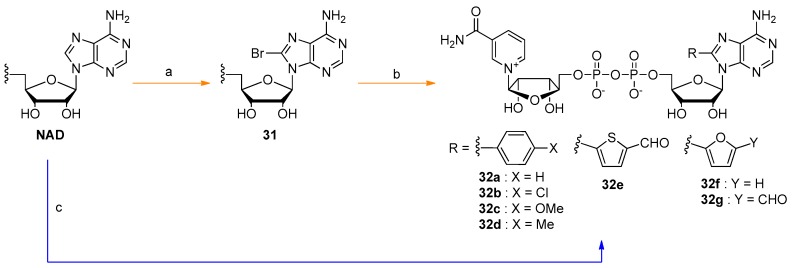
Synthesis of 8-substituted NAD^+^ analogs designed as inhibitors of sirtuins [60]. Conditions: a) (Br_2_)_aq_, (NaOAc)_aq_, pH 4, rt, 1.75 h; b) Na_2_Cl_4_Pd, TXPTS, R-B(OH)_2_, K_2_CO_3_, H_2_O, 40 °C, 20–35 min; c) i. neat Br_2_, (NaOAc)_aq_, pH 4, rt, 30 min; ii. Na_2_Cl_4_Pd, TXPTS, R-B(OH)_2_, K_2_CO_3_, H_2_O, 40 °C, 20–30 min.

In 2011, a set of 8-substituted NAD^+^ analogs as potential sirtuin inhibitors were designed by Pesnot et al. [60]. To avoid the most problematic step in NAD^+^ analog synthesis, which is pyrophosphate bond formation, the synthetic strategy was based on a one-pot 2-step sequence enabling direct functionalization of NAD^+^ (Scheme 2). The first step consisted of 8-selective bromination of NAD^+^ using saturated bromine water (orange path, Scheme 2) or neat bromine (blue path, Scheme 2), affording the intermediate **31**, followed by a key step of Suzuki-Miyaura cross-coupling reaction to introduce different substituents at the C8-position of adenine. The synthesized NAD^+^ analogs **32a**–**g** were then studied as inhibitors of SIRT1 and SIRT2.

All the 8-substituted NAD^+^ analogs showed inhibitory activity at low micro-molar concentrations toward SIRT2, but only moderate activity toward SIRT1. These first results suggested that the modification at the position 8 of NAD^+^ is generally well accepted by the SIRT1 enzyme. In further studies, only adenosine or adenosine monophosphate scaffolds, with or without the 8-modification, were tested toward the two sirtuins. It resulted in either a complete loss of activity, or in similar activity towards both enzymes, showing the importance of the complete NAD^+^ pattern for enzyme selectivity. NMR-based experiments directed at determining the conformation of the NAD^+^ analogs in solution provided hints about probable reasons for selectivity. The chemical shifts of the H2″ atoms indicated that all of the 8-substituted analogs adopt preferentially the “syn” conformation, whereas sirtuins would require the “anti” conformation in the active site, suggesting that the SIRT2 is more capable of inducing this conformational change than SIRT1. 

In 2012, Szczepankiewicz et al. [61] synthesized 4′-carba-NAD^+^ **33**, a stable and unreactive mimic of NAD^+^, in order to determine the ternary structures of SIRT3 and SIRT5 in cofactor-analog bound state. Because the natural NAD^+^ forms a short-lived complex with these sirtuins, using this 4′-carba-NAD^+^ was of great interest as it could not be subjected to nicotinamide displacement.

**Scheme 3:**
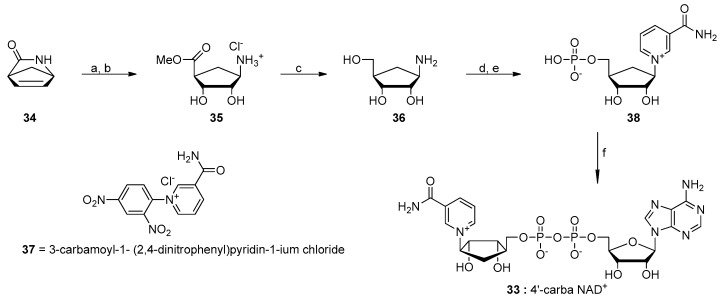
A synthetic pathway to 4′-carba-NAD^+^ (**33**) [61]. Conditions: a) i. OsO_4_, NMO, *t*-pentyl alcohol, H_2_O, 70 °C; b) MeOH, HCl, 25 °C; c) i. LiEt_3_BH, THF, 0 °C; ii. HF_(aq)_, 0 °C; iii. LiOH_(aq)_, 25 °C; d) **37**, NaOAc, MeOH, 25 °C; e) POCl_3_, (MeO)_3_PO, 0 °C; f) MnCl_2_, AMP-morpholidate, *p*-TsOH, formamide.

Although the synthesis of 4′-carba-NAD^+^ was described earlier [62], a new improved route was developed here. The synthesis was achieved in six steps, without requiring any protecting groups (Scheme 3). After the dihydroxylation and acidic methanol treatment of the lactam **34**, the resulting aminoester **35** was transformed in the amino derivative **36.** Then the glycosidic bond was formed by reaction with the derivative **37**, affording the 4′-carba NMN **38**. Finally, the latter was coupled with AMP morpholidate in the presence of pyridinium tosylate as a buffering agent in order to form the target dinucleotide **33**. 

The compound was then successfully used for the determination of the X-ray crystal structure of cofactor-analog bound SIRT3 in complex with its substrate protein, acetyl-CoA enzyme synthase 2 (ACS2). In this structure, a well-defined electron density from 4′-carba-NAD^+^ was visible, which made multipoint interactions with SIRT3 (Figure 7C). Interestingly, the conformation of the 4′-carba-NAD^+^ in this complex was very similar to the one in a previously determined ternary complex between yeast sirtuins yHst2, histone H4 peptide and 4′-carba-NAD^+^. The X-ray structure of the complex of SIRT5 with 4′-carba-NAD^+^ and isocitrate dehydrogenase 2 (IDH2), which is known to be dessuccinylated by SIRT5, revealed a very close similarity between complexes with SIRT3 and SIRT5 (Figure 7D). One difference is that 4′-carba-NAD^+^ formed more interactions with SIRT5 than with SIRT3, which is in agreement with the observation from authors that the *K*_m_ for the natural co-factor is lower for SIRT5 than for SIRT3. By comparing the structure of this complex with the one previously reported between SIRT5, succinyl H3 peptide, and NAD^+^, they have shown that both natural NAD^+^ and carba-NAD^+^ **33** adopt the same position in the different complexes. 

**Scheme 4:**
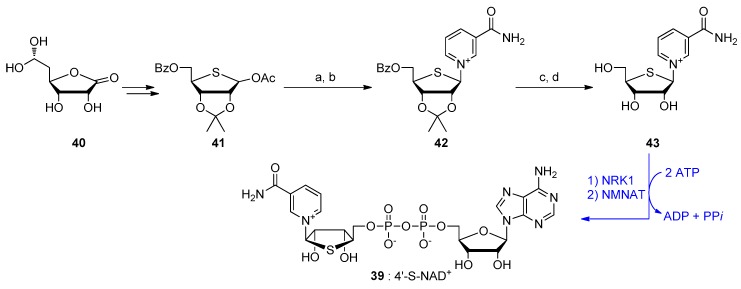
Chemoenzymatic pathway leading to the S-NAD^+^ **39** [63]. The enzymatic transformations are marked in blue. Conditions: a) HBr, toluene, 0 °C, 4 h; b) nicotinamide, MeCN, rt, 18 h; c) TFA/H_2_O, 0 °C, 6 h; d) NH_3_/MeOH, 0 °C, 48 h.

In 2018, Dai et al. [63] reported the chemoenzymatic synthesis of 4′-S-NAD^+^ **39**–an NAD^+^ analog that, similar to 4′-carba-NAD^+^, is stable against enzymes that cleave the C1′-N1 glycosidic bond, but features ribose geometry and electrostatic properties more similar to the native cofactor. The synthesis of the 4′-S-NAD^+^ has been performed starting from d-gluconic acid γ-lactone **40**, leading in eight steps to the thioribose **41** [64] (Scheme 4). Then, the introduction of the nicotinamide nucleobase afforded the protected compound **42**, followed by the removal of all the protecting groups leading to the nicotinamide 4′-thioribose **43**. Finally, a two-step enzymatic conversion using nicotinamide riboside kinase 1 (NRK1) and NMNAT led to 4′-S-NAD^+^.

The 4′-S-NAD^+^ was subjected to functional characterization in various NAD-related processes. As expected, the compound showed resistance to the N-glycosidic bond cleavage catalysed by CD38, inhibited this enzyme, and was successfully applied for X-ray structure determination of the complex (Figure 7B). 4′-S-NAD^+^ also inhibited activity of SIRT2. On the other hand, due to structural and electronic resemblance to NAD^+^, the analogue readily supported redox reactions, opening the opportunity for dissecting various NAD-dependent processes in complex biological systems.

NAD^+^ analogs have also been applied for the inhibition of ADP-ribosyltransferases. Langelier et al. [65] used an unhydrolyzable benzamide adenine dinucleotide (BAD) to study the auto-inhibition mechanism of PARP1. Functional and structural studies using this compound showed that auto-inhibition results from a selective disruption of NAD^+^ binding and revealed the existence of a reverse allosteric regulatory mechanism. 

### 4.2. NAD Analogs Targeting NAD-Dependent Metabolic Pathways

NAD-dependent redox reactions are key steps in many metabolic pathways, found across all living organisms. However, these pathways may differ between humans and pathogens, as well as between healthy and cancerous human cells, offering the chance for the development of selective inhibition strategies. Some of the crucial pathways that have been targeted by NAD^+^ analogs include the biosynthesis of nucleic acids, fatty acids, proteins, as well as the biosynthesis and transformations of NAD^+^ itself. 

#### 4.2.1. NAD Analogs Targeting Purine Biosynthesis Pathway

**Figure 9:**
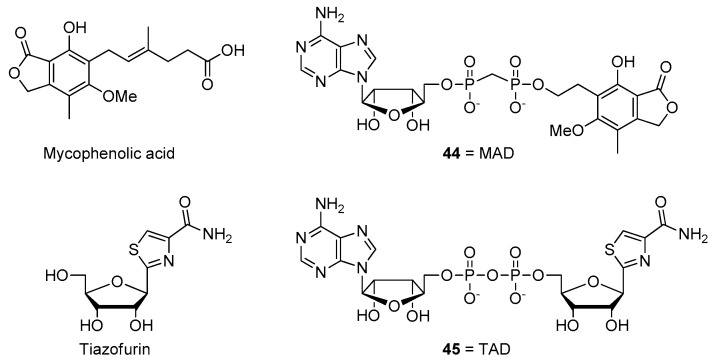
Some currently known inhibitors of IMPDH used in clinic and their derivatives [66].

Inosine-5′-monophosphate dehydrogenase (IMPDH) is an NAD-dependent enzyme responsible for guanine nucleotide de novo synthesis, necessary for the growth of cells and, thereby, is a target for the treatment of cancer, autoimmune diseases, and bacterial infections. There are two isoforms of human IMPDH, the *h*IMPDH1 and *h*IMPDH2, which share 84% sequence similarity and have similar activity, but differ in expression levels among cells and tissues. *h*IMPDH1 functions in angiogenesis, DNA-binding, and translation regulation. *h*IMPDH2 is usually up-regulated in proliferating cells, including cancer cells. Some IMPDH inhibitors have been tested in or have already entered the clinic (Figure 9). IMPDH is also necessary for de novo synthesis of guanine nucleotides in bacteria and, hence, constitutes an attractive antimicrobial target.

Pankiewicz and co-workers have developed a significant number of NAD^+^ analogs as potent inhibitors of IMPDH. This team has been working on IMPDH inhibitors for a few decades, and have published comprehensive reviews on the topic [66,67]; here, we will focus on their most recent works. Generally, NAD^+^ analogs can be designed to interact with three different subsites of the IMPDH cofactor binding domain: the nicotinamide binding subsite (N-subsite), the adenosine binding subsite (A-subsite), and the pyrophosphate binding subsite (P-subsite). Pankiewicz and coworkers have set out their work based on the structure of two of the four known IMPDH inhibitors, which are used in clinic (Figure 9) [66]. The first one is the mycophenolic acid (MPA), a nanomolar inhibitor of *h*IMPDH1, which blocks tumor-induced angiogenesis in vivo by interaction with the N-subsite, and is used as an immunosuppressant. This has led to the synthesis of MAD **44** as a potent inhibitor. The second is tiazofurin, biotransformed in cells in thiazole 4-carboxamide adenine dinucleotide (TAD, **45**), which is one of the most potent inhibitors of *h*IMPDH2 interacting with all three subsites and which is used in the treatment of chronic myelogenous leukemia (CML). It has to be noted that the ability of NAD^+^ derivatives to cross cell membranes is required for in vivo activity. Here, the replacement of the phosphate bridge by a methylene (MAD, 44) or its absence (tiazofurin) increases permeability of the derivatives. 

**Figure 10:**
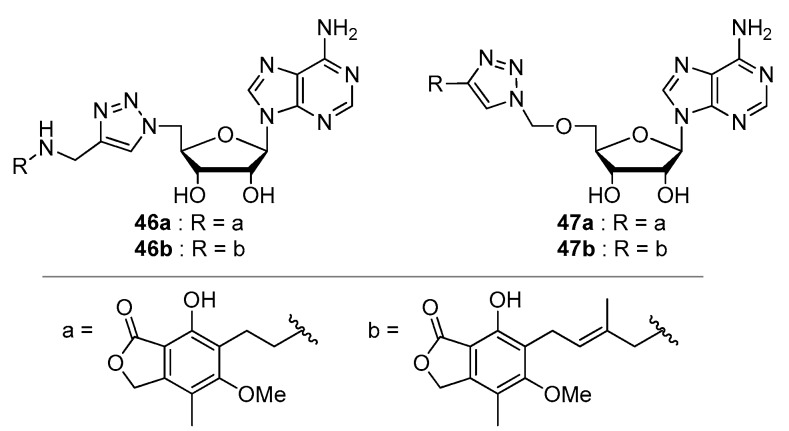
Triazol containing NAD^+^ analogs synthesized as potent inhibitors **46**–**47** [68].

In 2010, following their previous work on the synthesis of NAD^+^ analogs containing mycophenolic moiety and/or triazole link (Figure 10), Chen et al. [68] published a set of triazole-containing NAD^+^ analogs and focused on their inhibition activity toward the two isoforms of *h*IMPDH as well as *Mycobacterium tuberculosis* IMPDH (*Mtb* IMPDH), a potential target for the treatment of tuberculosis. They synthesized four compounds (**46a**–**b** and **47a**–**b**, Figure 10) by attaching mycophenolic moiety, as a replacement of nicotinamide, to adenine moiety through a 1,2,3-triazole linker. The latter has been introduced either directly at the 5′-position of adenosine (**46a**–**b**) or via an oxymethylene linkage (**47a**–**b**). The mycophenolic moiety has been linked to the triazole via either a C2 or a C4 chain.

The authors assessed the inhibitory activity toward *h*IMPDH1, *h*IMPDH2, and *Mtb*IMPDH. A better activity towards human enzymes was observed when the longer carbon chain was present (R = b), which has a similar length as the natural NAD^+^ substrate. The two compounds containing the C4 chain (**46b** and **47b**) showed similar nanomolar inhibition towards both *h*IMPDH1 and *h*IMPDH2, while the compounds with shorter carbon chain (**46a** and **47a**) displayed only micromolar activity. Interestingly, only the compound with a longer chain and containing the oxymethylene linker (**47b**) had a significant inhibition activity towards *Mtb*IMPDH. In order to understand the differences in activity, the authors used a previously obtained NAD^+^ analogue (MAD **44**, Figure 9) as a model for binding in the presence of inosine monophosphate (IMP), with the three IMPDHs. Computational studies combined with the X-ray crystal structure of the complex of NAD^+^ analog/IMPDH/IMP (*h*IMPDH2, Figure 7F) revealed aromatic interactions between adenine ring and enzyme for both human IMPDH, whereas no aromatic stacking was observed for *Mtb*IMPDH. Moreover, a hydrogen bond was present in the N-subsite binding domain only with human IMPDH, where an unfavorable binding was calculated for *Mtb*IMPDH. These differences in binding therefore explain the inhibition selectivity toward human IMPDH. Interestingly, similar computations performed using the new synthesized NAD^+^ analog (**47b**) revealed new favorable interactions in the P-subsite domain in addition to the absence of unfavorable ones, oppositely to the observations obtained when using the previous NAD^+^ analog. The lack of interactions in the A-subsite domain was, therefore, overcome thanks to these new compounds, and led to the first potent inhibitor of *Mtb*IMPDH. 

**Figure 11:**
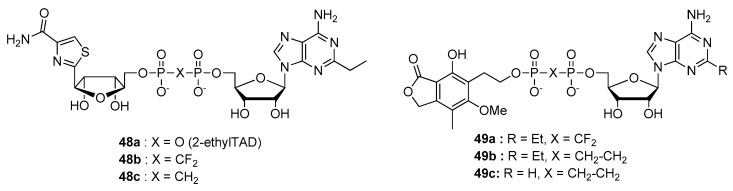
Potent inhibitors of IMPDH and K562 cell proliferation obtained by pyrophosphate linkage modification in TAD and MAD analogs [69].

Later, a new set of NAD^+^ analogs that are potential anticancer agents was described [69]. The authors mainly focused on attaching mycophenolic acid moiety to adenosine through a variety of linkers to improve the cellular permeability of compounds compared to those carrying a pyrophosphate (Figure 11). The resulting analogs were tested toward *h*IMPDH 1 and 2 and as inhibitors of leukemia K562 cells proliferation. When the pyrophosphate of 2-ethyl TAD **48a** was replaced by a difluoromethylenebisphosphonate (**48b**), it preserved the low nanomolar inhibition of *h*IMPDH 1 and 2, as well as increased the ability to penetrate cell membrane, resulting in micromolar inhibition of K562 cells proliferation (IC_50_ = 4.7 µM). In a similar way, 2-ethyl MAD analog with the same pyrophosphate modification (**49a**), improved its inhibitory activity toward *h*IMPDH1 and 2 (IC_50_ = 5 and 24 µM, respectively) compared to unmodified MAD **44**, and displayed a potent inhibition of K562 cells proliferation (IC_50_ = 0.45 µM), which made it the most potent inhibitor of K562 cells proliferation described so far.

Further development of ethylene bis(phosphonate) analogs to improve cell permeability led to even better inhibition of K562 cell growth (compounds **49b** and **49c**, IC_50_ = 4 and 10.5 µM, respectively). Phosphonophosphate (X = OCH_2_) containing compounds, despite being potent inhibitors of *h*IMPDH in vitro, showed low inhibitory effects on cells proliferation. Other modifications such as bis(sulfonamide), phosphonoacetamide, thioethylenephosphonate or triazole linkers also showed inhibitory activity, revealing that neither the tetrahedral geometry of phosphate nor the presence of phosphorus atom itself are necessary to maintain the inhibitory activity. Overall, the study showed that the P-subsite is a promising domain to introduce modifications, which can afford potent new inhibitors of IMPDH in vitro and in vivo. 

In 2011, the same group focused on the synthesis of NAD^+^ analogs with pyridone modification [70]. The study was inspired, among others, by the fact that in the 1970s, 4-pyridone-3-carboxamide was found in urine from patients suffering of chronic myelogenous leukemia (CML), becoming a potential marker for this disease [71]. Likewise, the corresponding triphosphate (4-pyridone-3-carboxamide 5′-triphosphate) was later found in high concentration in erythrocytes of patients with chronic renal failure (CRF). Therefore, the authors focused on the synthesis of NAD^+^ analogs, which contain a pyridone modification at different positions of the nicotinamide moiety, as well as a methylene bisphosphonate, instead of a pyrophosphate to improve compound stability and cell permeability. 

**Scheme 5:**
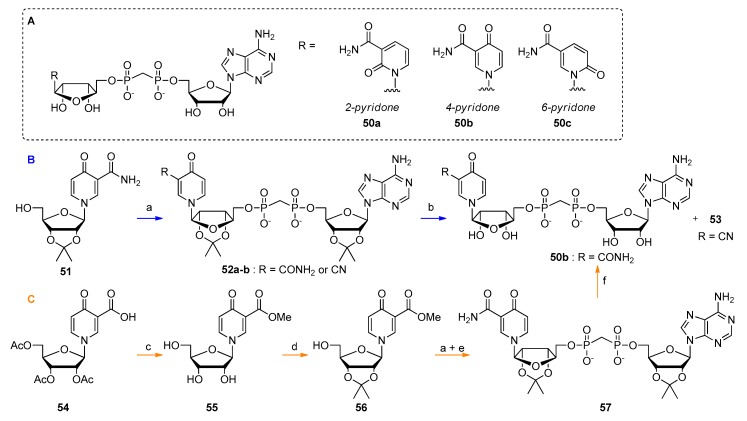
(**A**) Structure of the three NAD analogs carrying a pyridone moiety; (**B**,**C**) synthetic pathways developed for the pyridone containing NAD analogs [70]. Conditions: a) i. AMPCH_2_P, pyridine, DIC, rt, overnight, ii. nucleoside **51**, 70 h, 65 °C, iii. H_2_O, TEA, 6 h, 65 °C; b) 80% TFA, 3 h, 0 °C; c) i. NH_3_ in MeOH, 48 h, rt; ii. MeOH, TMSCH_2_N_2_ then AcOH, 15 min, rt; d) dimethoxypropane, acetone, *p-*TsOH, 3 h, rt; e. NH_3_ in MeOH, 3 days, rt; f) HCO_2_H 50%, overnight, rt.

To that end, three NAD derivatives (Scheme 5; 2-pyridone **50a**, 4-pyridone **50b** and 6-pyridone **50c**) were synthesized through a coupling reaction between 5′-adenosine methylenebisphosphonate and an appropriate isopropylidene-protected nucleoside in the presence of *N,N*-diisopropylcarbodiimide (DIC). The synthesis was first performed following the pathway B as represented with the 4-pyridone **50b** (Scheme 5B, blue pathway), from the corresponding pyridone **51**. Unfortunately, when applied to the carboxyamide derivatives, the coupling step led to the cyano derivatives (**52b** in the case of 4-pyridone) as a by-product. It was regrettably impossible to separate the by-product **53** from the desired compound **50b** by HPLC. Therefore, they developed another synthetic pathway (Scheme 5C, orange pathway) from the carboxylic acid derivative **54**, a strategy which has also been applied to the 2-pyridone derivative **50a**. Here, after the deprotection of acetyl groups and a methylation step affording the derivative **55**, the 2′,3′-hydroxyls were protected with an isopropylidene. The resulting compound **56** was subsequently coupled with the adenosine moiety leading to the protected NAD analog **57** before a last step of removal of the 2′,3′-protecting group. The 6-pyridone-containing analog was obtained following the first pathway, because in this case, the by-product and the desired compound could be separated by HPLC.

The compounds were subsequently tested towards *human* and *Mycobacterium* IMPDH and NAD kinase. Although no inhibition was found toward IMPDH, 6-pyridone NAD **50c** showed equal inhibition toward both *human* and *MtB* NAD kinase (same IC_50_ = 0.5 mM) and 2-pyridone **50a** was 8 times more potent inhibitor of *h*NADK than *Mtb*NADK. Interestingly, they also found that 4-pyridone-3-carboxamide-adenine dinucleotide phosphate is an inhibitor of *Toxoplasma gondi FNR* enzyme (*K*_i_ = 30 μM). 

**Figure 12:**
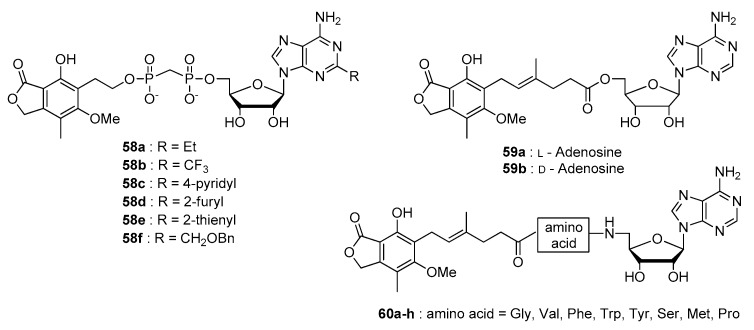
Three classes of MPA containing NAD analogs synthesized by Felczak et al. [72].

In 2014, the same group described three new classes of potent inhibitors of IMPDH [72], all based on previously described MAD **44** (Figure 9) [68]. The exploration of various adenosine modifications and replacements of the pyrophosphate moiety with more lipophilic groups led to the development of a second generation of IMPDH inhibitors (Figure 12). 

The first group of analogs carried a substituent at the C2 position of adenosine (**58a**–**f**). A set of six corresponding MAD derivatives was obtained by coupling different 2-substituted adenosines and mycophenolic bis(phosphonate). In vitro evaluation revealed compounds with high selectivity toward *h*IMPDH 1. In particular, the 4-pyridyl analog **58c** was 23 times more selective for *h*IMPDH 1 than 2 and was so far the most potent inhibitor of *h*IMPDH1 (*K*_i_ 0.6 nM). The compounds showed also a significantly improved antiproliferatory activity towards cancer cells (leukemia K562, Hela cervical cells, and colon cancer HT29 cells). The crystal structure of NAD^+^ bound to *h*IMPDH2 (Figure 7E), which suggested a preference for a strong aromatic system in A sub-domain, led to another MAD derivative wherein adenosine was replaced by 4-amino-benzimidazole. As expected, this compound had a slight preference for *h*IMPDH 2, but with only three times more potent inhibition activity. The second class of MAD-derived inhibitors was obtained by replacing the pyrophosphate with various esters linkers. The new MAD derivatives were synthesized both from l and d–adenosine (**59a**–**b**). They both showed an inhibition activity toward *h*IMPDH 1 and 2, but the d-ester was relatively stable toward esterases, whereas the l-ester was quickly cleaved (few minutes). An attempt to replace the ester by amide, expected to be resistant to esterases, led to potent inhibitor of IMPDH. Unfortunately, the compound did not show any anti-proliferative activity, likely due to its incapacity to cross cell membranes. The last class of MAD derivatives explored in this work was amino acid diamide derivatives, in which an amino acid is placed between the ‘adenosine amide’ moiety and mycophenolic acid moiety, via an extra amide link (**60a**–**h**). Both the final compounds containing different amino acids as well as all the precursors of the synthetic pathway (free acids, methyl esters and adenosine derivatives) were tested as inhibitors of *h*IMPDH 1 and 2 and anti-proliferative agents. In general, the final MAD derivatives had nanomolar activity in vitro and were more potent than the corresponding precursors, but they did not show anti-proliferative activity, probably because they were not able to penetrate the cells. Overall, the results underlined how even minor structural alterations may dramatically influence the biological activity of the studied inhibitors. 

#### 4.2.2. NADK Targeting Nucleotides and Pronucleotides

The NMN/Nicotinic acid mononucleotide (NaMN) adenylyl transferase and the NAD kinase are key enzymes involved in NAD metabolism. The first class of enzyme transforms NMN/NaMN into the corresponding NAD/NaAD using ATP. It is present in humans as three different isoforms, *h*NMNAT 1, 2 and 3, the first one is located in nucleus, whereas isoforms 2 and 3 are present in the Golgi complex and in mitochondria, respectively. The NAD kinase (NADK) is responsible for the 2′-O-phosphorylation of NAD(H), leading to NADP(H), using ATP or other sources of active phosphate. This enzymatic reaction is the only known pathway for the formation of NADP(H) in both eukaryotic and prokaryotic cells. Three different classes of NADK activity can be distinguished regarding its substrate specificity. The first one is represented by the human ATP-NAD kinase, which utilizes only ATP as a phosphoryl donor. The second one, found in gram positive bacteria, is ATP/polyphosphate NAD kinase, where inorganic polyphosphate can be used as an alternative to ATP. The third one is NADK activity from gram-negative bacteria and single cellular eukaryotes, named as NTP-NADK because of its ability to use as a substrate NTPs other than ATP. The discovery of bacterial NADK activities, in particular, the enzymes from *Mycobacterium tuberculosis* and *Listeria monocytogenes* [73] made this NAD kinase an attractive antibiotic target. Moreover, human NADK is also a therapeutic target for cancer [74]. The therapeutic potential of NMNAT and NADK inhibitors has been reviewed in 2011 [75].

**Figure 13:**
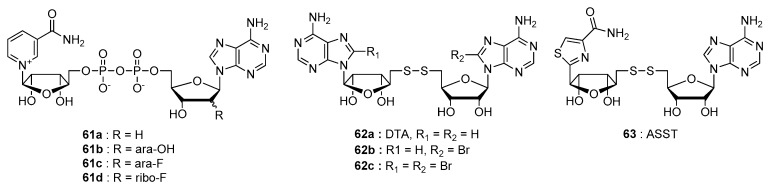
NAD-derived inhibitors of NADK [76].

The first identified inhibitors of NADK are NdAD (**61a**, Figure 13) and NdADH [75], which both lack the 2′-hydroxyl group, making the phosphorylation step impossible. Based on this fact, Petrelli et al. proposed other ribose 2′-modifications, namely an inversion of configuration of the hydroxyl (**61b**) or the replacement of the hydroxyl by a fluorine substituent, in both *arabino* (**61c**) and *ribo* (**61d**) configuration [76]. The simple inversion of configuration was enough to block the phosphorylation step; however, all of these modifications led to rather weak inhibitory activity of *human* and *Mtb* NADK. A known NAD analog, the benzamide adenine dinucleotide (BAD), was also assessed and found to be a potent inhibitor of *h*NADK (*K*_i_ 90 µM). Based on the earlier findings of Poncet-Montagne et al. [73], who published the crystal structure of *Listeria monocytogenes* NADK in both apo and NAD(P) bound state, and described di-5′-thio-adenosine (DTA **62a**) as a potent inhibitor (*K*_i_ 20 µM), the authors also tested this compound against *human* and *Mtb* NADK. This analog **62a** was a moderate inhibitor of these enzymes, with an IC_50_ of 45 µM and 87 µM, respectively. Consequently, they performed the synthesis of disulfide NAD analogs. Based on the structure of DTA and tiazofurin, which is a known inhibitor of IMPDH that is transformed in cells into TAD **45**, the adenosine was replaced by tiazofurin to yield ASST (**63**) and TSST. While the first one showed quite potent inhibition of *human* and *Mtb* NADK (Ki= 110 µM and 80 µM, respectively), the di-tiazofurin compound was inactive, revealing the importance of at least one adenosine moiety. 

Following these results, they next introduced a bromine atom on the 8 position of adenosine of DTA, forming either mono (**62b**) or di-substituted (**62c**) derivatives. Indeed, it has been described that this substituent can block the “syn” conformation of the corresponding compound, which could increase the inhibitory potency if preferred by the enzyme. In this case, both mono and dibromo compounds were found to inhibit *h*NADK (IC_50_ = 6 µM for both) and *Mtb*NADK (IC_50 =_ 19 µM and 14 µM, respectively) in a similarly potent way. Thereby, it can be assumed that this enzyme has indeed a preference for the *syn* conformation. On the other hand, compounds modified by introducing a methyl group at either the 2′- or 3′-position on both ribose moieties in order to fix the sugar conformation showed a weaker inhibition of both *human* and *Mtb*NADK than the unmodified parent compound (DTA, **62a**) or even no activity at all (3′-methyl derivative). 

**Figure 14:**
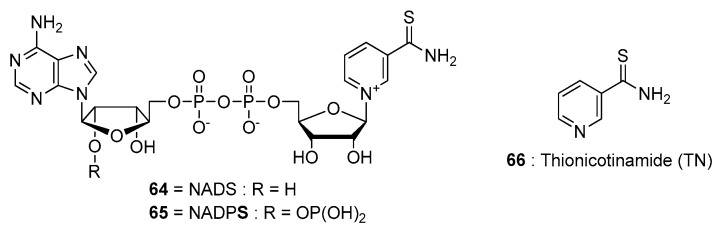
NAD analogs studied as NADK inhibitors inducing DHFR degradation [77] and G6PD inhibition [78].

The group of Bertino and coworkers explored the anticancer properties of NADK inhibitors. This was in contrast to previous studies that, except for a few examples [75,76], focused mainly on infectious diseases. Since NADPH plays a crucial role in the synthesis of nucleic acids and lipids, cancer cells require it for growth and proliferation. The deficiency of NAD(P)H can result in inhibition of tumor cells growth or even induce their death. NADPH, as a reducing agent, is also responsible for the regulation of reactive oxygen species (ROS) levels, wherein moderate levels of these species are beneficial for cancer cells, while excess induces apoptosis. In 2013, Hsieh et al. [77] reported the degradation of dihydrofolate reductase (DHFR) as a result of NADK inhibition by a newly identified NADK inhibitor, thionicotinamide adenine dinucleotide phosphate (NADPS). The DHFR is an NADP^+^ dependent enzyme playing a crucial role in DNA and RNA synthesis. Thus, the inhibition of DHFR leads to the inhibition of the nucleic acids synthesis, and thereby to cell death, making it an important therapeutic target for cancer. Notably, DHFR is the molecular target for methotrexate (MTX)—a drug widely used in cancer treatment. Because NADK is the only enzyme producing cellular NADP(H) from NAD(H), the authors envisaged that its inhibition should result in decrease of the NADPH pool, necessary for DHFR stability, and consequently inducing DHFR degradation. The authors assessed 14 different potential inhibitors of human cytosolic NADK starting with benzamide riboside. Although this compound showed cNADK inhibition in vitro, and therefore decreased NADPH levels, toxicity in animals was revealed by in vivo tests. Among the other 13 NAD(P) analogs tested, only the two compounds, thionicotinamide adenine dinucleotide and its 3′-phosphate (NADS **64** and NADPS **65,** respectively, Figure 14) were potent inhibitors of NADK (ED_50_ 1 µM). The equal effectiveness of both compounds was explained by occurrence of 3′-phosphorylation of NADS to NADPS by NADK, designating in both cases the phosphorylated form as the active one.

The inhibitors were next tested on two different cell lines, a metastatic human colon cancer cell line (C85 cells), and T-cell lymphoblastic leukemia cells. First, it was found that the inhibition of NADK by NADPH is nicotinamide dose dependent in a reverse manner. Next, by comparing C85 cells and normal cells, it was confirmed that NADPS exhibits a selective activity toward cancer cells. Additionally, by testing the effect of NADPS on three different dehydrogenases (GADPH, IMPDH and ALDH1a) in C85 cells, they observed that there was no change in levels of these enzymes, confirming its specificity for NADK/DHFR. Further, the molecular mechanism behind the decrease in DHFR level induced by NADPS was studied. Neither DHFR transcription nor translational upregulation was involved in DHFR decrease, confirming that lowering NADPH pool by NADK inhibition is responsible for this observation. Indeed, DHFR is stabilized by NADPH and depletion of NADPH makes this protein more susceptible to degradation. Finally, a synergistic cell death effect was observed when NADPS was combined with methotrexate, suggesting that NADK inhibition could be a promising way to overcome methotrexate-resistance. A model for NAD(P)^+^ complex with NADK was also developed, revealing that NADP^+^ shows less stable binding than NAD^+^, but the binding of NADPS^+^ is more favorable to a higher number of stabilizing van der Waals interactions. 

In 2015, Tedeschi et al. [78] studied the effect of thionicotinamide (TN **66**, Figure 14) on cytosolic NADPH pool, expecting it to both inhibit NAD-dependent metabolic pathways and impair the control of ROS species, leading to cell death. They showed that TN acted as a pronucleotide penetrating efficiently into cells and being transformed into NADS and NADPS, which inhibited NADK and glucose 6-phosphate dehydrogenase (G6PD). They also showed that nicotinamide lowers the toxicity of TN in C85 cells, indicating that TN selectively targets NAD-related pathways. Treatment of C85 cells with 100 μM TN resulted in NADK inhibition and decrease in NAD(P)H pool overtime (up to 60–70% in less in 24 h). Additionally, a significant depletion of fatty acids and inhibition of protein synthesis were observed. TN alone had no significant influence on ROS levels, but in combination with oxidative stress (H_2_O_2_), the ROS levels drastically increased, suggesting loss of control on ROS levels. Similar results were obtained when using TN simultaneously with either a chemotherapeutic drug (gemcitabine, docetaxel, irinotecan) or menadione, all known to generate ROS. TN did not show neurotoxicity in mice xenograft model. It is an improvement compared to 6-aminonicotinamide, a known G6PD inhibitor, which showed severe neurotoxicity during anti-cancer clinical trial. This study further affirmed NADK as a promising target for cancer treatment.

#### 4.2.3. Miscellaneous and Unidentified Molecular Targets

**Figure 15:**
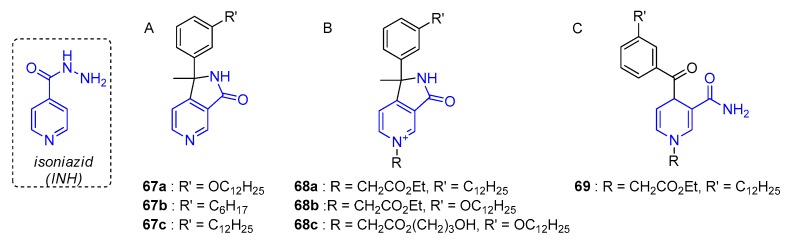
Three classes of isoniazid-nicotinamide adduct analogs developed as inhibitors of InhA from *Mycobacterium tuberculosis*: A - pyridine, B - pyridinium, C - dihydropyridine; and structure of the most relevant inhibitors [79,80].

NAD analogs with biological activity have also been synthesized for other molecular targets or without a specific target in mind. A series of adducts of an anti-turberculosis drug, isoniazid (INH, Figure 15), with truncated NAD moiety modified with a lipophilic hydrocarbon chain was developed by Delaine et al. [79]. Indeed, it has been reported earlier that the active form of isoniazid creates INH-NAD adduct, which inhibits the enoyl-ACP reductase (InhA) enzyme from *Mtb*, leading to the block of fatty acid elongation. Thereby the biosynthesis of the mycolic acid, essential compound of the mycobacteria envelop is stopped. However, resistance to INH has been increasingly observed, and can be explained either by a mutation in *Mtb*, which decreases the ability of INH to form a complex with NAD or by a mutation of the enzyme, which interacts with diphosphate moiety of NADH. Therefore, the authors designed and synthesized a set of compounds with modified and truncated INH-NAD moiety, to overcome the resistance coming from the diphosphate moiety. The first generation of truncated INH-NAD analogs reported in a preceding work did not afford sufficient inhibition of InhA. Hence in this work, in order to enhance the affinity and the selectivity for InhA catalytic site, a lipophilic chain was added to the nicotinamide pattern using Suzuki-Miayura cross-coupling reaction. To that end, three different classes of nicotinamide derivatives (pyridines **67**, pyridinums **68** and dihydropyridine **69**, Figure 15) were prepared and assessed as InhA and bacterial growth inhibitors. As a result, five of these compounds (**67a, 68a**–**c, 69,** Figure 14) were identified as potent InhA inhibitors. 

In their next paper, another set of those derivatives (compounds **67a**–**c, 68a, 69**) were tested against *Corynebacterium glutamicum*, which does have InhA activity, *Escherichia Coli* (as possessing an InhA homolog, it would test mycobacteria selectivity) and *Plasmodium Falciparum* (malaria pathogen agent) [80]. The pyridine **67a** inhibited specifically *Mtb*/ *Mycobacteria smegmatis* (*Ms)* and not *C. glutamicum*, in the same way as INH, indicating that biological activity of this compound arises solely from InhA inhibition. In contrast, two other compounds (**68a** and **69**) inhibited both *Mtb*/*Ms* and *C. glutamicum*, proving that InhA enzyme is not the only one targeted by the compounds.

**Figure 16:**
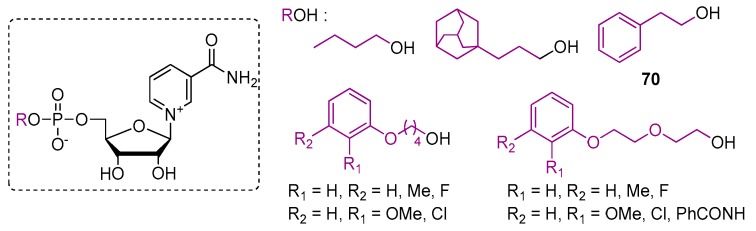
Phosphodiester analogs tested as bacterial and yeast growth enhancers, including the most potent compound **70** [81].

In 2009, a new method to synthesize nicotinamide-containing phosphodiester analogs was developed by Liu et al. [81]. They designed a set of fourteen NMN derivatives (Figure 16) to provide tools for biological studies of NAD-dependent processes. The analogs were functionalized within the phosphate group with an alkane or an aromatic substituent linked to the nicotinamide moiety by a phosphodiester bond. The synthesis was based on a key reaction between the nicotinamide monophosphate and the corresponding alcohol. These products were additionally 2′- and 3′-O acetylated to afford better solubility and higher reactivity. Among all the reagents tested, 2,4,6-triisopropylbenzenesulfonyl chloride (TIPS-Cl) was the most effective as a condensation agent and was used under optimized conditions. Preliminary biological assays showed that compound **70** (100 µM) had a beneficial effect on the growth of *Eschericia Coli* (DH5α) and *Saccaromyces cerevisiae* (ATCC 26108). The compound has no effect on the activity of *S. cerevisiae* alcohol dehydrogenase.

NaADP is acting as a second messenger to stimulate Ca^2+^ release. This process has been proved in many human systems, but the details of molecular mechanism behind it are still unraveled. Therefore, to study the NaADP-mediated control of Ca^2+^ release, the need of NaADP analogs is required for further biological studies as affinity binding experiments or labelling through a click reaction. To this end, the substitution of the 4 and 5-position of the nicotinic acid was explored by Jain et al. [82]. They revealed that small 5-substituents as methyl, ethyl, azido or amino were tolerated, whereas the 4-substitution led to agonists with low potency.

**Figure 17:**
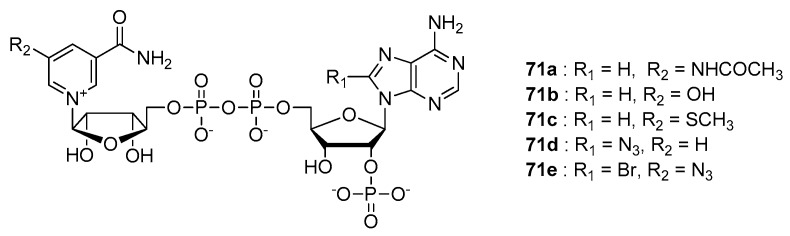
The most potent agonist-type NAD^+^ analogs developed by Trabbic et al. [83].

In a more recent work, Trabbic et al. [83] designed a set of 11 new analogs, carrying modification at the 5 position of the nicotinic acid and/or at the 8-position of adenosine (Figure 17). After the assessment of spectroscopic properties, they evaluated ability of the compounds to affect three different processes from *Strongylocentrotus purpuratus* (sea urchin egg, a model of NaADP-mediated Ca^2+^ release in mammals). Firstly, they checked their aptitude of releasing Ca^2+^ using a fluorometric assay where the Ca^2+^ release is linked to an increase in fluorescence. A competition ligand binding assay revealed that the analog efficiently competed with ^32^P-NaADP. Finally, an experiment evaluating the ability of the compounds to induce subthreshold receptor desensitization was performed. As a result, the 5-substituted derivatives with small substituents were identified as highly potent agonists (**71a**–**c**, Figure 17). Moreover, they showed that 8-subsituted compounds, in particular the 8-azido **71d** and the disubstituted derivative **71e** also displayed potent agonist activity at low concentration. 

## 5. NAD Analogs as Alternative Redox Co-Factors

NAD^+^ analogs can also be considered as alternative or even biorthogonal co-factors for cellular redox processes. Such analogs can aid better understanding of enzymatic reactions and provide new hydride donors/acceptors useful for enzymatic or chemical transformations. This topic has been quite recently reviewed by Paul and co-workers [84]; therefore, here we will only highlight a few select examples. 

**Figure 18:**
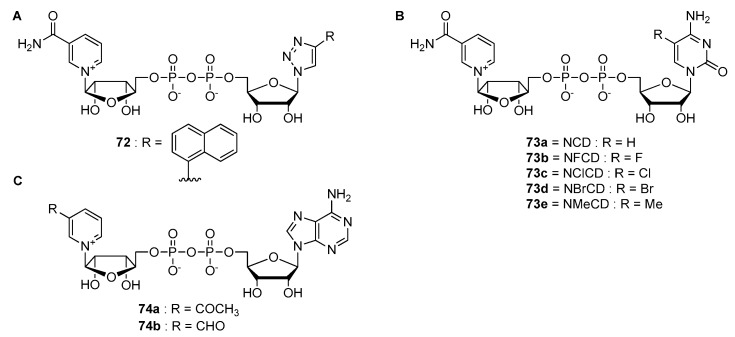
NAD^+^ analogs developed as cofactors for orthogonal redox systems. (**A**) Triazole derivative **72** [85,86]; (**B**) NCD and analogs [87,88,89]; (**C**) Nicotinamide modified compounds [90].

In 2011, Hou et al. [85] designed 13 NAD^+^ analogs, where the adenosine moiety was replaced by a 1,2,3-triazole moiety (Figure 18A). The ability of the compounds to be used as cofactors was then tested and revealed activity in the presence of malic enzyme and alcohol dehydrogenase, suggesting flexibility in the NAD-binding pockets of those enzymes. Later, by screening those analogs against different mutants of malic enzyme, they identified a mutant (ME-L30K/L404S) characterized by over 1000-fold preference for analog **72** over NAD^+^ [86]. Thus, the study laid foundation for the development of an orthogonal redox system with potential for metabolic pathway engineering.

Ji et al. [87] focused on the development of NCD and analogs (Figure 18B, compounds **73a**–**e**). NCD **73a** and NFCD **73b** were found to display high activity as cofactors for a particular mutant of malic enzyme (ME-L310R/G401C). They later developed other analogs, NClCD **73c**, NBrCD **73d** and NMeCD **73e** [88], which in the same way showed good orthogonality for the natural pair ME/NAD^+^, except for the last one (NMeCD). Recently [89], NAD^+^ analog **73a** was also found to be used as a non-natural cofactor by the phosphite dehydrogenase (Pdh) mutants, revealing a preference for NCD over NAD.

Another class of analogs was developed by Lee et al. [90], where the amide group of the nicotinamide moiety of NAD was replaced by different substituents (Figure 18C). These derivatives were used as artificial electron carriers for photo-enzymatic synthesis under visible light. The study revealed that the compounds **74a** and **74b** were the most efficient reducing agents.

## 6. NAD-Derived Chemical Tools to Study NAD-Capped RNAs

In eukaryotic cells, the 5′ end of mRNA is protected with 7-methyl guanosine (m^7^G) via a 5′-5-triphosphate bridge. More recently, it has been discovered that both in bacteria and in higher organisms, including yeast, mammalian cells, and plants, NAD^+^ is present at the 5′-end of some RNAs [9,12,14,15,17,91,92]. However, the biogenesis and biological functions of this RNA modification are still unraveled. To better understand its role, several groups have worked on this newly discovered structure, to provide a way for the chemical synthesis of NAD-RNA models [93], to create methods for detection and quantification of NAD-capped RNAs in cells [9,94,95], and to modulate its susceptibility to different enzymes [96]. 

### 6.1. Chemoenzymatic Methods of NAD-RNA Detection 

Since the discovery of NAD-capped (or NAD-linked) RNA, several groups have been working on new ways to detect it in cells, since the initially established MS-based method is time-consuming and requires large amounts of RNA [17]. A widely-adopted chemo-enzymatic method for identification of NAD-capped RNAs taking advantage of the ADP-ribosyl cyclase (ADPRC) activity was developed by Cahova et al. [9]. The method involves a substitution of the terminal nicotinamide moiety in NAD-RNA by an alcohol with terminal alkyne group through ADPRC action. Only RNAs terminated with 5′-NAD^+^ undergo this transformation. The resulting alkyne-conjugated RNA is then subjected to a click reaction with biotin azide. The last step consists of isolation of biotinylated RNAs on streptavidin beads and the consequent analysis of the RNA by high throughput sequencing. 

More recently, Vvedenskaya and co-workers [94] developed CapZyme-Seq method to detect and quantify not only NAD-capped RNA but also other non-canonical initiating nucleotides (NCIN), as FAD- and dpCoA- capped RNAs. The first step is the processing of the RNA using NudC or Rai1 enzyme, both releasing 5′-P-RNA by either a pyrophosphatase or a phosphodiesterase activity, respectively. Then a high throughput sequencing is used to quantify the capped-RNA.

NAD-capQ is another method for the detection and quantification of NAD-RNAs developed by Grudzien-Nogalska et al. [95]. NAD-capQ is a two-step procedure, which involves enzymatic treatment of the RNA with nuclease P1, resulting in the phosphodiester bonds cleavage, followed by the quantification of the released 5′-NAD^+^ through a commercially available colorimetric assay. Thus, the method does not require any specialized laboratory equipment (except for a simple spectrophotometer) and is rapid in comparison to the previous method, but does not provide information on the sequences of the quantified RNAs.

### 6.2. Synthesis of Molecular Tools to Study NAD-Capped RNAs 

**Figure 19:**
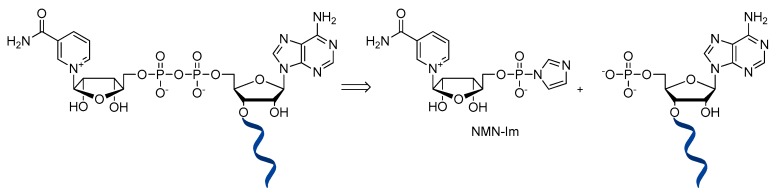
NAD-RNA synthesis strategy according to Hofer et al. [93].

Since this area of research has emerged quite recently, not many NAD-derived tools to study NAD-RNAs have been developed so far. The first chemical synthesis of NAD capped RNA was described by Hofer et al. [93], as an alternative to enzymatic synthesis using in vitro transcription (IVT) [97]. The IVT method requires the use of NAD^+^ in large excess and leads to a mixture of NAD- as well as 5′-triphosphate RNA, which are difficult to separate. Hofer et al. [93] used either an 5′-P-RNA 20 mer obtained by solid phase synthesis or 38 mer prepared from in vitro transcription, followed by pyrophosphatase treatment to afford the 5′-monophosphate from 5′-triphosphate (Figure 19). 

To add NMN moiety to these RNAs, they synthesized nicotinamide 5′-phosphorimidazolide by activating NMN using CDI in DMF followed by a carbonate hydrolysis step. In the last step, they used 1000× excess of the resulting NMN-Im in coupling reaction with 5′-P-RNA in an aqueous media containing Mg^2+^. This step led to the mixture of NAD-RNA and unreacted 5′-P-RNA. This latter was however easily removed by treatment with Xrn-1, a nuclease which uses only 5′-P-RNA as substrates. The authors used the same method to afford the nicotinamide guanosine (NGD), uridine (NUD) and cytidine (NCD) dinucleotide-capped RNAs. They also showed that the synthetic NAD-RNA, similarly as the one obtained by IVT, is accepted by the deNADding enzyme, NudC–a Nudix hydrolase responsible for removal of the NAD^+^ moiety from RNA in bacteria.

In 2018, our team reported on the synthesis of novel NAD^+^ analogs and their incorporation into RNA [96]. The goal of the study was to provide NAD-RNA analogs that are less susceptible to so-far-identified deNADding enzymes than the native structure. Several enzymes capable of removing the 5′-NAD^+^ from RNA were identified. Generally, they can be divided into two different classes: (i) deNADding enzymes with pyrophosphatase activity (e.g., NudC and *h*Nudt12) and (ii) deNADding enzymes with phosphodiesterase activity (e.g., Rai or DXO) [10]. The first group cleaves the pyrophosphate bond in NAD-RNA, releasing the nicotinamide 5′-monophosphate and the 5′-P-RNA, whereas the second group removes the entire NAD^+^ moiety from RNA 5′ also revealing 5′-P-RNA.

**Figure 20:**
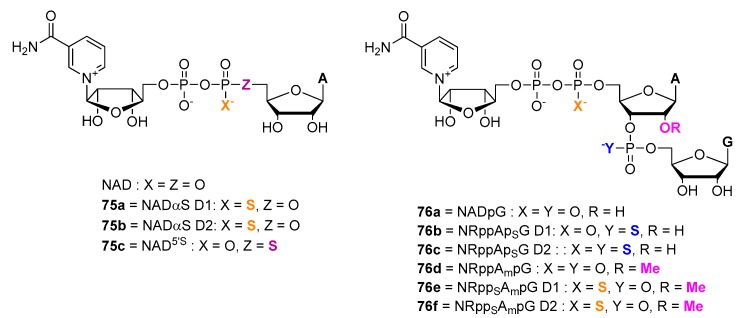
Hydrolysis-resistant NAD^+^ analogs designed to study the corresponding NAD-RNAs [96].

Taking into account the cleavage sites of these enzymes, we designed a set of NAD^+^ analogs–either di- or trinucleotides–containing modifications on the pyrophosphate bridge, 2′-position of adenosine and/or within the phosphodiester bond between NAD^+^ and the third nucleotide (Figure 20). The synthesis was performed using a ZnCl_2_-mediated coupling reaction between NMN-Im and an appropriate adenosine-containing mono- or dinucleotide. The compounds have later been incorporated into RNA via IVT using polymerase T7 and a 10-fold excess of NAD^+^ analog, followed the analysis by polyacrylamide gel electrophoresis to determine RNA quality and NAD^+^ analog incorporation efficiency. Remarkably, it was found that trinucleotide NAD^+^ analogs **76a-f** are very efficiently incorporated into RNA compared to NAD^+^ or dinucleotide NAD^+^ analogs **75a-c**. The unmodified trinucleotide (NADpG, **76a**) is therefore a superior reagent for generation of NAD-capped RNAs by IVT compared to NAD^+^. 

The resulting NAD-RNAs were subjected to three different deNADding enzymes, which led to the identification of compounds showing resistance only to deNADding pyrophosphatases (NAD^5′S^, **75c**), only to phosphodiesterase (NRppA_m_pG, **76d**), or both types of activities (NRpp_S_A_m_pG D1 **76e** and NRpp_S_A_m_pG D2 **76f**). The corresponding NAD-RNA analogs are expected to be more stable under cellular conditions and therefore have a potential to become useful molecular tools for the studies on its biological role of NAD-RNA.

## 7. Conclusions and Perspectives for the Future

Here, we reviewed the recent progress in the design of NAD analogs as molecular probes and biologically active compounds. The last decade has brought many significant advancements in the design of fluorescent probes enabling real-time monitoring of NAD-dependent processes via fluorescent methods. Although most of these probes have been utilized only in vitro or in cell extracts*,* the first impressive applications in living cells have also been achieved [45]. We envisage that the field will progress further in this this direction, especially if universal solutions for efficient intracellular delivery of NAD analogs are developed. A step in this direction was reported recently by Wang et al. [98], who developed an *Escherichia coli* strain expressing an efficient NAD importer and showing limited extracellular degradation to facilitate characterization of NAD analogs in vivo. If similar solutions are also devised for eukaryotic cells, they will greatly advance NAD-related research in complex biological settings. These solutions could also facilitate probe and inhibitor design by helping dissect issues with compound permeability form other problems with in vivo activity. NAD-derived inhibitors have undoubtedly tremendous potential for the treatment of cancer, bacterial infections, age-related disorders (the latter via targeting of sirtuins), and other diseases. However, in this context, the selectivity of the NAD-derived inhibitors is a very important, yet often concealed issue. Many structurally similar compounds have been designed with completely different molecular targets in mind and showed activity in quite unrelated biological systems. It is difficult to point out substitution sites that are selectively preferred by particular enzyme classes as for most of them systematic structure-activity relationship studies have not yet been completed. One of the exceptions are ADPribosyltransferases (e.g., PARP) for which it has been determined that the preferred modification sites are N6 and C2 positions of adenine and 3′-*O* position of nicotinamide riboside. In the case of other NAD-related enzymes, adenine modifications at the N6 and C8 positions have been commonly explored (among others) and often proved to be biologically active. Taking into account the overlapping artificial substrate and inhibitor scopes for many enzymes, it is likely that some of the observed discrepancies between the results from in vitro and in vivo evaluation arise from off-target effects. Standardization of inhibitor evaluation procedures, preceded by identification of enzyme families that have similar structural requirements for NAD recognition, could help addressing the issue of selectivity at early stages of inhibitor development. Finally, the discovery of NAD-capped RNA has opened completely new horizons for NAD analog development. Here, of great value may be compounds and methods enabling assaying the activity of enzymes responsible for NAD-RNA biosynthesis and degradation in vitro and in vivo, compounds enabling capture and isolation of specifically interacting proteins from biological mixtures, and inhibitors selectively targeting proteins that bind or process NAD-capped RNA. 

## Figures and Tables

**Table 1:** Optical properties of NAD and select adenosine-modified emissive NAD analogs.

Compound	Modified Nucleoside	λ_ex_^a^	λ_em_^a^ (Φ)	Enzymatic Activities Tested with NAD Analog ^b^	Refs.
NAD^+^		259	-	n/a	[31]
NADH		340	460 (0.02)		
ε-NAD^+^ 1	1,N6-Ethenoadenosine (ε-A)	300	415	Glutamate dehydrogenase (+) NAD glycohydrolase (8%) NAD pyrophosphatase (54%) Alcohol dehydrogenase (3%) PARP (-)	[40,41,42]
8-(Pyr)NAD^+^ 2e	8-(2-pyrrolyl)adenosine	300	410 (0.005)	NAD glycohydrolase (+) nucleotide pyrophosphatase (+) ADPR cyclase/cADPR hydrolase (+)	[32]
N^tz^AD^+^ 6	isothiazolo[4,3-d]pyrimidine-riboside (^tz^A)	336	411 (0.038)	Alcohol dehydrogenase (+) NAD glycohydrolase (+) Mono-ADP-ribose transferase (ART 5^c^/CTA^d^) (+) NAD^+^ kinaseEnzymatic synthesis from ^tz^ATP by NMNAT (+) PARP (+/-)	[33,38,39]
N^tz^ADH		336	412 (0.015)	Lactate dehydrogenase (+) NAD glycohydrolase (+)	
N^th^AD^+^ 8	thieno[3,4-d]pyrimidine-riboside (^th^A)	341	431 (0.071)	Enzymatic synthesis from ^th^ATP by NMNAT (+) Alcohol dehydrogenase (+) NADase (+) Mono-ADP-ribose transferase (CTA) (+) PARP (-)	[39]

^a^ λ values are given in nm; ^b^ values in brackets indicate relative activity reported in reference to NAD; “+” indicates that the co-factor supported enzymatic activity comparably to NAD or at an analytically useful level; “-” indicates that the co-factor did not support the enzymatic activity, the reaction was very inefficient or analytically not useful. ^c^ Hydrolysis to ADPR was observed as the major process; ^d^ ADP-ribosylation was observed as the major process.; n/a–not applicable.

## Abbreviations

AAC	Azide alkyne cycloaddition
Ach	Acetylcholine
ACS	Acetyl-coA enzyme synthase
ADH	Alcohol dehydrogenase
ADPRC	ADP-ribosyl cyclase
ALDH	Aldehyde dehydogenase
Aq	aqueous
ART	ADP-ribosyl transferase
ASST	Tiazofurin adenosine disulfide
BAD	Benzamide adenine dinucleotide
cADPR	Cyclic ADP-ribose
CDI	1,1′-carbonyldiimidazole
CML	Chronic myleogenous leukemia
CRF	Chronic renal failure
CTA	Cholera toxin subunit A
CuAAC	Copper catalysed AAC
dpCoA	Dephospho coenzyme A
DHFR	dehydrofolate reductase
DIC	*N,N*-diidopropylcarbodiimide
DMF	Dimethylformamide
DMSO	Dimethylsulfoxide
DTA	di-5′-thioadenosine dinucleotide
FAD	Flavine adenine dinucleotide
FLIM-FRET	Fluorescence lifetime imaging microscopy–Förster resonance energy transfer
G6PDH	Glucose-6-phosphate dehydrogenase
GADPH	Glyceraldehyde phosphate dehydrogenase
GFP	Green fluorescent protein
IDH	Isocitrate dehydrogenase
IEDDA	Inverse electron demand Diels-Alder cycloaddition
IMP(DH)	Inosine-5′-monophosphate (dehydrogenase)
INH	Isoniazid
InhA	Enoyl-ACP reductase
IVT	in vitro transcription
LDH	Lactate dehydrogenase
MAD	Mycophenolic acid adenine dinucleotide
MART	MonoADP-ribosyl transferase
MeCN	Acetonitrile
MPA	Mycophenolic acid
*Mtb*	Mycobacterium
MTX	Methotrexate
NaAD	Nicotinic acid adenine dinucleotide
NAD	Nicotinamide adenine dinucleotide
NADase	NAD glycohydrolase
NADK	NAD kinase
NAD(P)S	Thionicotinamide adenine dinucleotide (phosphate)
Nam	Nicotinamide
NaMN	Nicotinic acid mononucleotide
NaMNAT	NaMN adenylyl transferase
NCIN	Non-canonical initiating nucleotides
NCD	Nicotinamide cytidine dinucleotide
NdAD	Nicotinamide-2′-deoxy adenosine dinucleotide
NGD	Nicotinamide guanosine dinucleotide
NMN	Nicotinamide mononucleotide
NMNAT	NMN adenylyl transferase
NMO	4-methylmorpholine *N*-oxide
NRK	Nicotinamide riboside kinase
NUD	Nicotinamide uridine dinucleotide
PAR	Poly(ADP-ribose)
PARG	Poly(ADP-ribose) glycohydrolase
PARP/ARTD	Poly(ADP-ribose) polymerase
Pdh	Phosphite dehydrogenase
PP*i*	Pyrophosphate
ROS	Reactive oxygen species
Rt	Room temperature
SIRT	Sirtuin
SPAAC	Strain promoted AAC
TFA	Trifluoroacetic acid
TIPS-Cl	2,4,6-triisopropylbenzenesulfonyl chloride
^th^A	Thieno[4,3-d]pyrimidine riboside
TMR	Tetramethylrhodamine
TN	Thionicotinamide
TPPTS	3,3′,3″-Phosphanetriyltris(benzenesulfonic acid) trisodium salt
TSST	Ditiazofurin disulfide
TXPTS	Tris(2,4-dimethyl-5-sulfophenyl)phosphine trisodium salt
^tz^A	Isothiazolo[4,3-d]pyrimidine riboside
